# MARCH1 regulates insulin sensitivity by controlling cell surface insulin receptor levels

**DOI:** 10.1038/ncomms12639

**Published:** 2016-08-31

**Authors:** Arvindhan Nagarajan, Max C. Petersen, Ali R. Nasiri, Gina Butrico, Annie Fung, Hai-Bin Ruan, Romy Kursawe, Sonia Caprio, Jacques Thibodeau, Marie-Claude Bourgeois-Daigneault, Lisha Sun, Guangping Gao, Sanjay Bhanot, Michael J. Jurczak, Michael R. Green, Gerald I. Shulman, Narendra Wajapeyee

**Affiliations:** 1 Department of Pathology, Yale University School of Medicine, New Haven, Connecticut 06510, USA; 2 Howard Hughes Medical Institute, Yale University School of Medicine, New Haven, Connecticut 06510, USA; 3 Department of Cellular and Molecular Physiology, Yale University School of Medicine, New Haven, Connecticut 06510, USA; 4 Department of Internal Medicine, Yale University School of Medicine, New Haven, Connecticut 06510, USA; 5 Departments of Comparative Medicine, Yale University School of Medicine, New Haven, Connecticut 06510, USA; 6 Departments of Pediatrics, Yale University School of Medicine, New Haven, Connecticut 06510, USA; 7 Département de Microbiologie, Infectiologie et Immunologie, Université de Montréal, Montreal, Quebec Canada, H3T 1J4; 8 Gene Therapy Center, University of Massachusetts Medical School, Worcester, Massachusetts 01605, USA; 9 Isis Pharmaceuticals, Carlsbad, California 92008, USA; 10 Division of Endocrinology and Metabolism, Department of Medicine, University of Pittsburgh, Pittsburgh, Pennsylvania 15261, USA; 11 Howard Hughes Medical Institute and Department of Molecular, Cell and Cancer Biology, University of Massachusetts Medical School, Worcester, Massachusetts 01605, USA

## Abstract

Insulin resistance is a key driver of type 2 diabetes (T2D) and is characterized by defective insulin receptor (INSR) signalling. Although surface INSR downregulation is a well-established contributor to insulin resistance, the underlying molecular mechanisms remain obscure. Here we show that the E3 ubiquitin ligase MARCH1 impairs cellular insulin action by degrading cell surface INSR. Using a large-scale RNA interference screen, we identify MARCH1 as a negative regulator of INSR signalling. *March1* loss-of-function enhances, and *March1* overexpression impairs, hepatic insulin sensitivity in mice. MARCH1 ubiquitinates INSR to decrease cell surface INSR levels, but unlike other INSR ubiquitin ligases, MARCH1 acts in the basal state rather than after insulin stimulation. Thus, MARCH1 may help set the basal gain of insulin signalling. *MARCH1* expression is increased in white adipose tissue of obese humans, suggesting that MARCH1 contributes to the pathophysiology of T2D and could be a new therapeutic target.

## Abstract (web summary)

Insulin receptor levels at the cell surface are reduced in insulin resistance, for reasons that are not fully understood. Here, the authors identify the E3 ubiquitin ligase MARCH1 as a direct regulator of basal insulin receptor surface levels and, therefore, insulin signalling.

## Graphical abstract

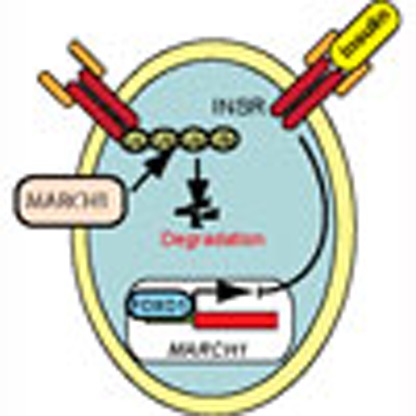

Insulin has potent metabolic and mitogenic effects that are mediated through binding to the insulin receptor (INSR)^1^. Dysregulated insulin signalling is central to the pathogenesis of the metabolic syndrome and type 2 diabetes (T2D), and is increasingly implicated in a variety of human cancers^2^. Insulin-resistant tissues require increased insulin secretion to produce physiological responses, and this compensatory hyperinsulinemia is deleterious both for the overextended β-cell and for its mitogenic effects on cancer cells^2^. An improved understanding of both normal insulin signalling and cellular insulin resistance will guide the development of new therapies targeting this axis.

Efforts to understand the molecular basis of insulin resistance have implicated multiple intracellular processes^3^. The strong association between ectopic lipid accumulation in liver and muscle and insulin resistance in those tissues has led to the hypothesis that bioactive lipid metabolites, such as diacylglycerol, ceramides and acylcarnitines, interfere with insulin signalling effectors^4^,^5^. Impaired INSR signalling in particular is a well-established defect in typical obesity-associated insulin resistance^6^. INSR dysregulation has two principal components: decreased INSR tyrosine kinase activity and decreased surface INSR content^7^,^8^,^9^. The former process has been linked to ectopic lipid accumulation through activation of PKCɛ, but cellular mechanisms mediating the latter process are incompletely understood^10^,^11^,^12^.

INSR dysregulation has profound effects on whole-body metabolism. Patients with *INSR* mutations (Donohue syndrome, Rabson–Mendenhall syndrome) exhibit major growth defects and insulin resistance so severe as to mimic untreated type 1 diabetes^13^. Rodent studies of global and tissue-specific INSR deletion have confirmed the severe consequences of impaired INSR function^14^. Together, these studies suggest that cellular regulators of the INSR itself, rather than downstream signalling effectors, may have particularly profound effects on cellular insulin signalling. Therefore, identification of such endogenous INSR regulators will aid efforts to understand the cellular regulation of insulin signalling and may reveal new therapeutic targets for the treatment of T2D and other pathologies related to aberrant insulin signalling.

Several E3 ubiquitin ligases are known negative regulators of insulin signalling. Both INSR and insulin receptor substrate (IRS) proteins are regulated by ubiquitination^15^,^16^,^17^,^18^,^19^. The canonical model for INSR ubiquitination involves insulin-dependent recruitment of the E3 ligase, facilitating endocytosis and endosomal sorting to attenuate signalling from the activated INSR^18^. CBL and NEDD4 are two ubiquitin ligases implicated in this process^18^,^20^,^21^. However, the complexity of the ubiquitin code—monoubiquitination, multimonoubiquitination and polyubiquitination can serve diverse functions and hundreds of E3 ligases are encoded in the genome—suggests that current understanding of the role of ubiquitination in insulin signalling is incomplete. In this regard, we hypothesized that systematic analysis of ubiquitin ligases would yield insights into cellular regulation of insulin signalling. Here, with the aim of identifying repressors of insulin signalling, we performed a large-scale RNAi screen targeting 616 human E3 ubiquitin ligases. Our large-scale RNAi screen identified MARCH1 as a potent and previously unstudied repressor of insulin signalling. Functional studies using multiple cell-based and mouse models revealed that MARCH1 is itself insulin-regulated, and that it is both necessary and sufficient for normal cellular control of insulin action. Further work established that MARCH1 acts by regulating surface INSR levels in the basal low-insulin state, tuning cellular insulin sensitivity. Notably, this mechanism differs from that of previously reported INSR ubiquitin ligases, which are activated only after insulin stimulation.

## Results

### RNAi screen identifies repressors of insulin signalling

To identify new repressors of insulin signalling, we carried out an unbiased, large-scale RNAi screen. To perform the screen, we generated a lentiviral shRNA library containing a total of 2,833 shRNAs targeting 616 E3 ubiquitin ligases and their adapter proteins (Supplementary Table 1). The choice to target E3 ubiquitin ligases was guided by our recognition that despite the diverse and potent cellular functions of ubiquitination, the regulation of insulin signalling effectors by the ubiquitin code is incompletely understood but likely to participate heavily in the cellular control of insulin action.

The RNAi screen exploited the requirement of HeLa cells for high concentrations of insulin when cultured in serum-free medium (Supplementary Fig. 1a). HeLa cells exhibited intact insulin signalling, as indicated by robust insulin stimulation of AKT Ser^473^ phosphorylation (Supplementary Fig. 1b).

**Figure 1:**
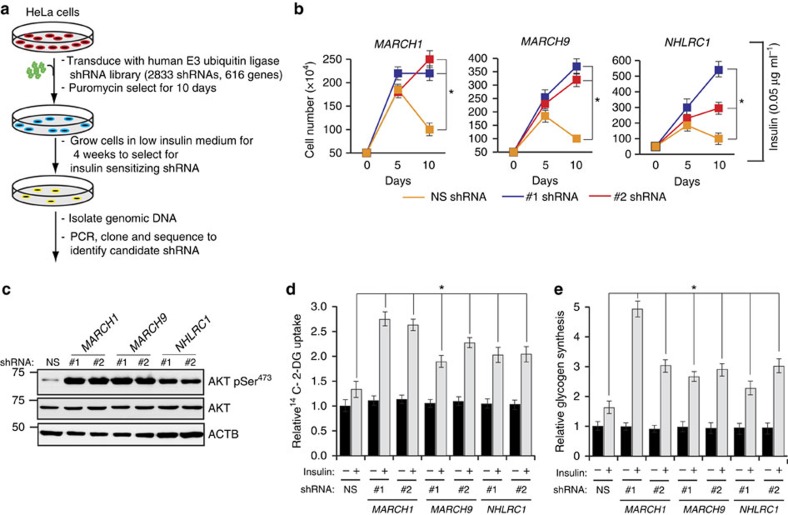
A large-scale RNAi screen identifies MARCH1 as a repressor of insulin signalling. (**a**) Schematic summary of the RNAi screen. (**b**) Relative cell number (*n*=3), calculated using Trypan blue exclusion, for HeLa cells expressing indicated shRNAs and grown in suboptimal insulin concentration (0.05 μg ml^−1^) for the indicated number of days. *compares NS and *MARCH1* shRNAs at day 10. (**c**) Immunoblot analysis of AKT Ser^473^ phosphorylation in HeLa cells expressing either NS or *MARCH1*, *MARCH9* or *NHLRC1* shRNAs. (**d**) Relative ^14^C-2-deoxyglucose uptake (*n*=4) in HeLa cells expressing indicated shRNAs. *compares NS and indicated shRNAs in presence of insulin. (**e**) Relative glycogen synthesis (*n*=3) in HeLa cells expressing indicated shRNAs. *compares NS and indicated shRNAs in presence of insulin. In all panels, data are mean±s.e.m. and *P<0.05, comparisons by *t*-test. NS, nonsilencing.

To begin the large-scale RNAi screen (Fig. 1a), we infected HeLa cells with three different pools of E3 ubiquitin ligase libraries in triplicate; each pool contained ∼1,000 shRNAs targeting ∼200 genes. Control cells were infected with a nonspecific shRNA (NS shRNA). After infection, cells were grown in suboptimal insulin concentrations (0.05 μg ml^−1^; 8.6 nM) insufficient to support HeLa cell growth and survival. Continued culture in suboptimal insulin concentrations thus provided selection pressure favouring clones whose shRNA conferred an insulin-sensitizing effect. After 4 weeks in suboptimal insulin, surviving colonies were collected and genomic DNA was isolated and sequenced to identify candidate genes. We recovered seven candidate repressors of insulin signalling, for which multiple shRNAs were identified by sequencing in all three replicates (Supplementary Table 2).

### Validation of candidate repressors of insulin signalling

After identifying the seven potential repressors of insulin signalling, we performed multiple assays to examine their role in insulin action. First, we individually knocked down all seven candidates using two sequence-independent shRNAs (Supplementary Fig. 1c). Knockdown of any of the seven candidates enabled robust proliferation in 0.05 μg ml^−1^ insulin, while control shRNA-expressing cells failed to proliferate (Fig. 1b and Supplementary Fig. 2a). This was not secondary to an insulin-independent mitogenic advantage, because no differences in proliferation were observed in 10% serum-supplemented medium (Fig. 1b and Supplementary Fig. 2b).

After this, we directly tested the effect of each candidate repressor on insulin signalling by stimulating serum-starved HeLa cells with 0.05 μg ml^−1^ insulin. Out of the seven candidates, shRNA-induced knockdown of three (*MARCH1*, *MARCH9* and *NHLRC1*) resulted in increased AKT Ser^473^ phosphorylation (Fig. 1c and Supplementary Fig. 2c), increased ^14^C-2-deoxyglucose (2-DG) uptake (Fig. 1d and Supplementary Fig. 2d), and increased glycogen synthesis (Fig. 1e and Supplementary Fig. 2e).

**Figure 2:**
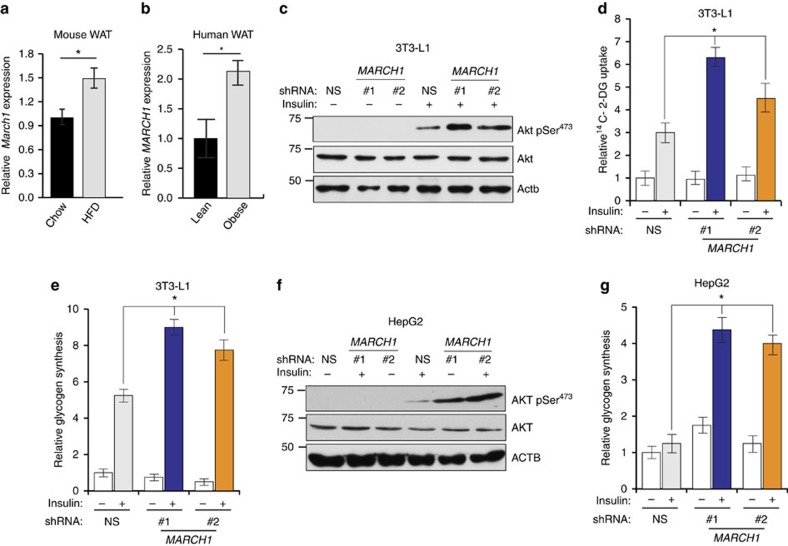
MARCH1 expression is increased in WAT from obese humans and mice and regulates insulin signalling in target tissues. (**a**) qRT-PCR measurement of *March1* expression in WAT of adult male C57BL/6 J mice fed either regular chow or high-fat diet. (**b**) qRT-PCR measurement of *MARCH1* expression in WAT from lean (*n*=8) and obese (*n*=13) human adolescent subjects. (**c**) Immunoblot analysis of AKT Ser^473^ phosphorylation in differentiated 3T3-L1 adipocytes expressing indicated shRNAs. (**d**) Relative ^14^C-2-deoxyglucose uptake (*n*=4) in differentiated 3T3-L1 adipocytes expressing either NS or *March1* shRNA. (**e**) Relative glycogen synthesis (*n*=3) in differentiated 3T3-L1 adipocytes expressing either NS or *March1* shRNA. (**f**) Immunoblot analysis of AKT Ser^473^ phosphorylation in HepG2 hepatocytes expressing either NS or *MARCH1* shRNA. (**g**) Relative glycogen synthesis (*n*=3) in HepG2 hepatocytes expressing either NS or *MARCH1* shRNA. In **d**,**e**,**g**, *compares NS and *MARCH1* shRNAs in presence of insulin. In all panels, data are mean±s.e.m. and *P<0.05, comparisons by *t*-test. qRT-PCR, quantitative real-time PCR.

Next, we investigated whether any identified candidates were physiologically relevant repressors of insulin signalling. Reasoning that increased expression of the candidate gene in insulin-resistant tissue could indicate that the gene contributes to insulin resistance, we examined whether mRNA expression of any candidate gene was upregulated in high-fat diet (HFD) fed, insulin resistant mice. Only *March1* expression was significantly upregulated by HFD in white adipose tissue (WAT) (Fig. 2a). On the basis of these results, we measured *MARCH1* expression in WAT biopsies from obese and lean human adolescents. Consistent with our results in HFD-fed mice, *MARCH1* expression was increased approximately twofold in WAT from obese humans (Fig. 2b). Further, microarray gene expression analysis of the same patient cohort stratified into insulin-sensitive and insulin-resistant groups revealed increased *MARCH1* expression in the insulin-resistant group (1.29-fold, *P*=0.006) (ref. 22). We therefore focused subsequent studies on MARCH1, which has been primarily studied in secondary lymphoid tissues^23^,^24^,^25^,^26^ and has not been previously linked to insulin signalling or metabolic regulation.

To test whether *MARCH1* knockdown could increase insulin action in physiologically relevant cell types, we employed 3T3-L1 adipocytes and HepG2 hepatocytes (Supplementary Fig. 2f–g). We found that insulin-stimulated AKT phosphorylation (Fig. 2c), 2-DG uptake (Fig. 2d) and glycogen synthesis (Fig. 2e) were significantly increased by *March1* knockdown in 3T3-L1 adipocytes. Similarly, HepG2 hepatocytes displayed enhanced insulin-stimulated AKT phosphorylation (Fig. 2f) and glycogen synthesis (Fig. 2g) after *MARCH1* knockdown.

### *March1* knockdown enhances insulin sensitivity in mice

**Figure 3:**
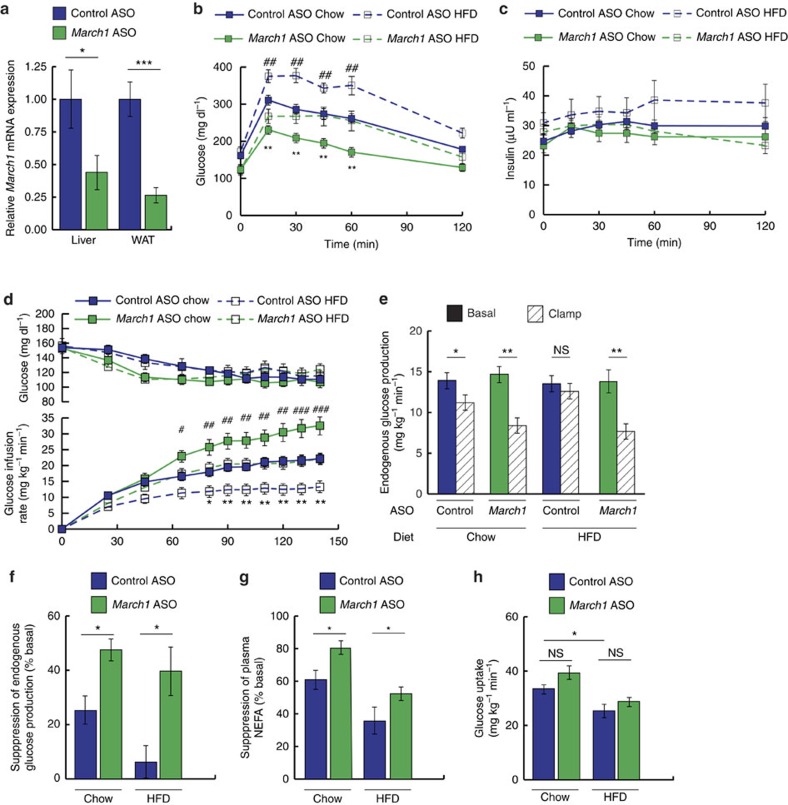
*March1* knockdown improves insulin sensitivity in mice. Male 12–20-week-old C57BL/6 J mice were treated with ASO for 2 weeks and fed regular chow or HFD as indicated. (**a**) Relative *March1* mRNA expression in liver and WAT. (**b**,**c**) Plasma glucose (**b**) and insulin (**c**) excursions during ipGTT. (**d**) Plasma glucose and glucose-infusion rates during hyperinsulinemic-euglycemic clamp studies in mice fed regular chow or 3 weeks of HFD and treated with *March1* ASO or control ASO. # denotes comparison of chow-fed groups and *denotes comparison of HFD-fed groups. (**e**) EGP during the basal period and during the steady-state period of the clamp. (**f**) Suppression of EGP during the clamp, expressed as % basal EGP. (**g**) Suppression of plasma NEFA concentrations during the clamp, expressed as % basal. (**h**) Whole-body glucose uptake during the clamp. Data are mean±s.e.m. In all panels, *P<0.05, **P<0.005, ^##^*P*<0.005, ***P<0.0005, ^###^*P*<0.0005. In **a**, *n*=3 mice per group; comparisons by two-tailed unpaired *t*-test. In **b**,**c**, *n*=5–8 mice per group; comparisons by two-way ANOVA. In **d**–**h**, *n*=9–11 mice per group; comparisons by two-way ANOVA. ANOVA, analysis of variance; ipGTT, intraperitoneal glucose tolerance tests.

To investigate the *in vivo* role of MARCH1 in insulin action, we first pursued a loss-of-function approach, treating mice with 2'-*O*-methoxyethyl chimeric antisense oligonucleotides (ASO) targeting *March1* mRNA or with a control ASO targeting no known mouse gene. *March1* ASO achieved transcript knockdown of ∼60% in liver and ∼80% in WAT after 2 weeks of treatment, with no knockdown in skeletal muscle or evidence of hepatotoxicity (Fig. 3a and Supplementary Fig. 3a–d). When challenged with HFD during the two weeks of ASO treatment, mice receiving *March1* ASO did not significantly differ in body weight but gained less fat mass than controls (Supplementary Fig. 3e–f).

During intraperitoneal glucose tolerance tests of both chow-fed and HFD-fed mice, *March1* ASO improved glucose tolerance without affecting insulin levels—suggesting improved insulin sensitivity rather than enhanced insulin secretion (Fig. 3b,c and Supplementary Fig. 3g,h). In chow-fed mice, the insulin-sensitizing effect of *March1* ASO was further enhanced by extending ASO treatment to 4 weeks; in addition to improved plasma glucose excursions, 4-week *March1* ASO-treated mice displayed significantly reduced insulin excursions (Supplementary Fig. 3i,j). To test whether the apparent insulin-sensitizing effect of *March1* ASO treatment could be attributed to increased energy expenditure or decreased caloric intake, we performed metabolic cage studies. These experiments revealed no effect of *March1* ASO on energy expenditure, locomotor activity, caloric intake, O_2_ consumption or respiratory quotient on either regular chow or HFD (Supplementary Fig. 3k–q).

Finally, we performed hyperinsulinemic-euglycemic clamp studies in awake mice to investigate tissue-specific effects of *March1* knockdown on insulin sensitivity. To increase our power to detect an insulin-sensitizing effect of *March1* knockdown on regular chow diet, we used a low insulin dose of 2.0 mU kg^−1^ min^−1^, expected to submaximally suppress endogenous glucose production (EGP) in control ASO treated, chow-fed mice. Plasma insulin levels were raised to similar levels during the clamp studies (Supplementary Fig. 3r). In both chow-fed and HFD-fed mice, *March1* knockdown increased the glucose infusion rate required to maintain euglycemia compared with controls, demonstrating improved whole-body insulin sensitivity (Fig. 3d and Supplementary Fig. 3s). Insulin suppression of EGP was enhanced by *March1* ASO approximately twofold on chow diet and approximately sixfold on HFD, indicating improved hepatic insulin action (Fig. 3e,f). In addition, insulin suppression of plasma non-esterified fatty acid (NEFA) levels was increased by *March1* ASO, reflecting enhanced insulin action in WAT (Fig. 3g). However, insulin-stimulated peripheral glucose uptake was not significantly altered, consistent with the tropism of the ASO (Fig. 3h). Together, these ASO studies offer *in vivo* evidence for negative regulation of liver and adipose insulin signalling by MARCH1 in both insulin-sensitive and insulin-resistant mice.

### *March1* ^*−/−*^ mice are insulin sensitive on both chow and HFD

**Figure 4:**
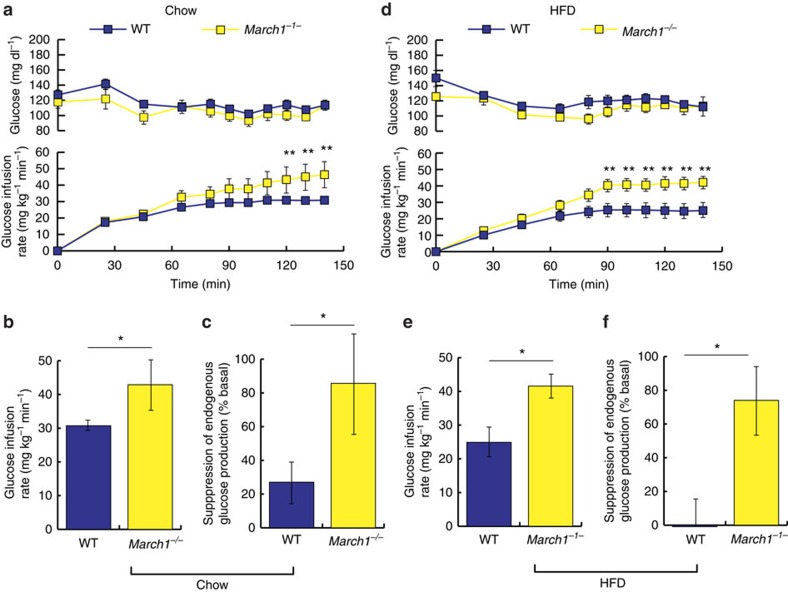
*March1*^*−/−*^ mice display improved hepatic insulin action. Littermate wild-type control mice were used in all studies of *March1*^*−/−*^ mice. Different insulin infusion rates were used for studies of chow-fed and HFD-fed mice (2 and 3 mU kg^−1^ min^−1^, respectively), so data are not superimposed. (**a**) Plasma glucose and glucose infusion rates during hyperinsulinemic-euglycemic clamp studies in male mice fed regular chow. (**b**) Mean steady-state glucose infusion rates in chow-fed male mice. (**c**) Insulin suppression of EGP in chow-fed male mice, expressed as % basal EGP. (**d**) Plasma glucose and glucose infusion rates during hyperinsulinemic-euglycemic clamp studies in male mice fed HFD for 3 weeks. (**e**) Mean steady-state glucose infusion rates in HFD-fed male mice. (**f**) Insulin suppression of EGP in HFD-fed male mice. Data are mean±s.e.m. In all panels, *P<0.05, **P<0.005. All comparisons by two-tailed unpaired *t*-test. In **a**–**c**, *n*=10 WT and five knockout mice per group. In **d**–**f**, *n*=5 mice per group.

To further investigate the effect of MARCH1 loss on insulin action in mice, we performed hyperinsulinemic-euglycemic clamp studies in a previously established line of *March1*^*−/−*^ mice^27^. On regular chow diet, age- and weight-matched male *March1*^*−/−*^ mice displayed decreased fasting plasma insulin but similar fasting glucose (Fig. 4a and Supplementary Fig. 4a,b). During hyperinsulinemic-euglycemic clamp studies, chow-fed *March1*^*−/−*^ mice required significantly greater glucose infusion rates to maintain euglycemia compared with wild-type littermate controls (Fig. 4a,b). This increase in whole-body insulin sensitivity was accounted for by improved EGP suppression; insulin-stimulated peripheral glucose uptake did not significantly differ between groups (Fig. 4c and Supplementary Fig. 4c,d). We also performed intraperitoneal glucose tolerance tests in female chow-fed *March1*^*−/−*^ mice, which revealed improved glucose tolerance with no difference in plasma insulin—suggestive of improved insulin sensitivity (Supplementary Fig. 4e–g). Next, to test whether *March1* deletion would confer protection from HFD-induced insulin resistance, we subjected *March1*^*−/−*^ mice to 3 weeks of high-fat feeding. *March1*^*−/−*^ mice displayed statistically insignificant trends toward lower body weight, adiposity and fasting plasma insulin (Supplementary Fig. 4h–j). During hyperinsulinemic-euglycemic clamp studies, HFD-fed *March1*^*−/−*^mice again required greater glucose infusion rates to maintain euglycemia, an effect accounted for both by improved EGP suppression and by a statistically insignificant trend towards increased peripheral glucose uptake (Fig. 4d–f and Supplementary Fig. 4k,l). Together, these data are consistent with the phenotype of *March1* ASO-treated mice and indicate that MARCH1 loss improves hepatic insulin action in mice.

### *March1* overexpression impairs insulin action in mouse liver

**Figure 5:**
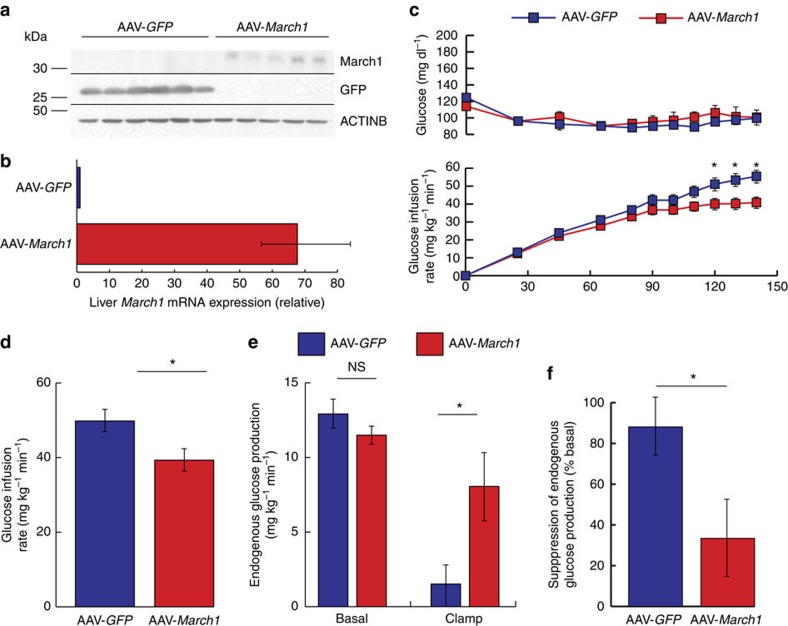
Ectopic *March1* expression is sufficient to impair hepatic insulin action in mice. Male 12-week-old C57BL/6 J mice were injected intravenously with AAV 4 weeks before study and fed regular chow. (**a**) Immunoblot analysis of GFP and March1 protein expression after AAV treatment. (**b**) qRT-PCR analysis of hepatic mRNA *March1* expression after AAV treatment. (**c**) Plasma glucose and glucose infusion rates during hyperinsulinemic-euglycemic clamp studies. (**d**) Mean steady-state glucose infusion rates required to maintain euglycemia during the clamp. (**e**) EGP during the basal period and during the steady-state period of the clamp. (**f**) EGP suppression. Data are mean±s.e.m. In all panels, *P<0.05, ***P<0.0005. *n*=7-8 mice per group; comparisons by two-tailed unpaired *t*-test. qRT-PCR, quantitative real-time PCR.

Next, to examine whether MARCH1 is sufficient to impair insulin action *in vivo*, we used an adeno-associated viral vector (AAV) to ectopically express *March1* or GFP control in liver. Mice were studied 4 weeks after a single-intravenous AAV dose. Despite a >65-fold increase in *March1* mRNA expression, March1 protein was barely detectable in liver lysates by immunoblotting (Fig. 5a,b)—consistent with its known post-translational autoregulation by autoubiquitination^23^,^26^,^27^,^28^. Consistent with our hypothesis, chow-fed mice receiving *March1* AAV required lower glucose infusion rates during hyperinsulinemic-euglycemic clamp studies (Fig. 5c,d and Supplementary Fig. 5a). This decrease was attributable to impaired EGP suppression rather than decreased peripheral glucose uptake (Fig. 5e,f and Supplementary Fig. 5b), indicating that ectopic MARCH1 expression is sufficient to impair insulin action in mouse liver. Body weight was similar between groups for the full duration of AAV treatment (Supplementary Fig. 5c).

### *MARCH1* transcription is repressed by insulin through FOXO1

**Figure 6:**
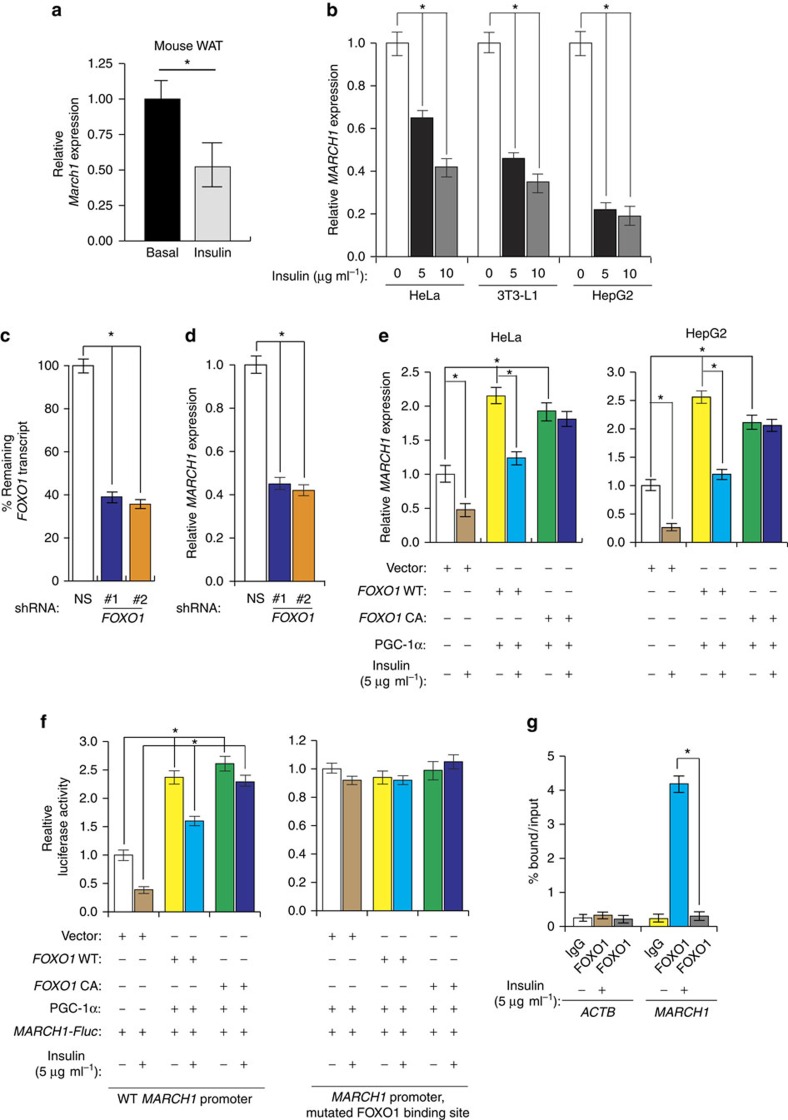
Insulin signalling represses *MARCH1* transcription through FOXO1. (**a**) qRT-PCR measurement of *March1* expression in wild-type mouse WAT after 6 h fasting (basal) or after 2 h of insulin stimulation (clamp). *n*=8 (basal) and 9 (clamp) mice per group. (**b**) qRT-PCR measurements of *MARCH1* expression (*n*=3) in the indicated cell lines after acute insulin stimulation at the indicated doses. (**c**) qRT-PCR measurement of *FOXO1* mRNA expression (*n*=3) in HeLa cells expressing either a NS or *FOXO1* shRNA. (**d**) qRT-PCR measurement of *MARCH1* mRNA expression (*n*=3) in HeLa cells expressing either a NS or *FOXO1* shRNA. (**e**) qRT-PCR measurements of *MARCH1* expression (*n*=3) in HeLa (left) or HepG2 (right) cells expressing empty vector, wild-type FOXO1, constitutively active FOXO1 and/or PGC-1α and treated with insulin as indicated. (**f**) Luciferase reporter assay (*n*=3) for wild-type *MARCH1* promoter (left) or a mutant *MARCH1* promoter with a mutated FOXO1-binding site (right) in HeLa cells expressing empty vector, wild-type *FOXO1*, constitutively active *FOXO1* (*FOXO1 CA*), and/or PGC-1α and treated with insulin as indicated. (**g**) ChIP measurement of FOXO1 protein enrichment on the *MARCH1* or *ACTIN* promoter with and without insulin treatment (*n*=3) as indicated. Data are mean±s.e.m. In all panels, *P<0.05, comparisons by *t*-test. qRT-PCR, quantitative real-time PCR.

We next returned to our original observation that *MARCH1* expression was increased in obese mouse and human WAT. Reasoning that *MARCH1* upregulation in insulin resistance would be consistent with loss of normal repression by insulin, we tested whether insulin regulates *MARCH1* expression. In mouse liver, we found that *MARCH1* expression was decreased by acute insulin stimulation (Fig. 6a). This downregulation was confirmed in cultured HeLa cells, HepG2 hepatocytes and 3T3-L1 adipocytes (Fig. 6b). Analysis of the *MARCH1* promoter and intergenic region revealed several putative FOXO1-binding sites, and shRNA-mediated knockdown of *FOXO1* in HeLa cells decreased basal expression of *MARCH1* (Fig. 6c,d). Similarly, ectopic expression of FOXO1 with its co-activator PGC-1α-induced *MARCH1* expression in HeLa and HepG2 cells, and expression of a constitutively active FOXO1 mutant abolished insulin regulation of *MARCH1* expression (Fig. 6e). A *MARCH1* promoter-luciferase reporter was activated by FOXO1/PGC-1α co-expression, and this effect required intact FOXO1-binding sites in the *MARCH1* promoter (Fig. 6f). Chromatin immunoprecipitation (ChIP) experiments confirmed that FOXO1 binds to the *MARCH1* promoter in the absence of insulin and this binding was inhibited upon insulin addition (Fig. 6g). Collectively, these results demonstrate that insulin represses *MARCH1* expression in a FOXO1-dependent manner.

### MARCH1 controls plasma membrane insulin receptor levels

**Figure 7:**
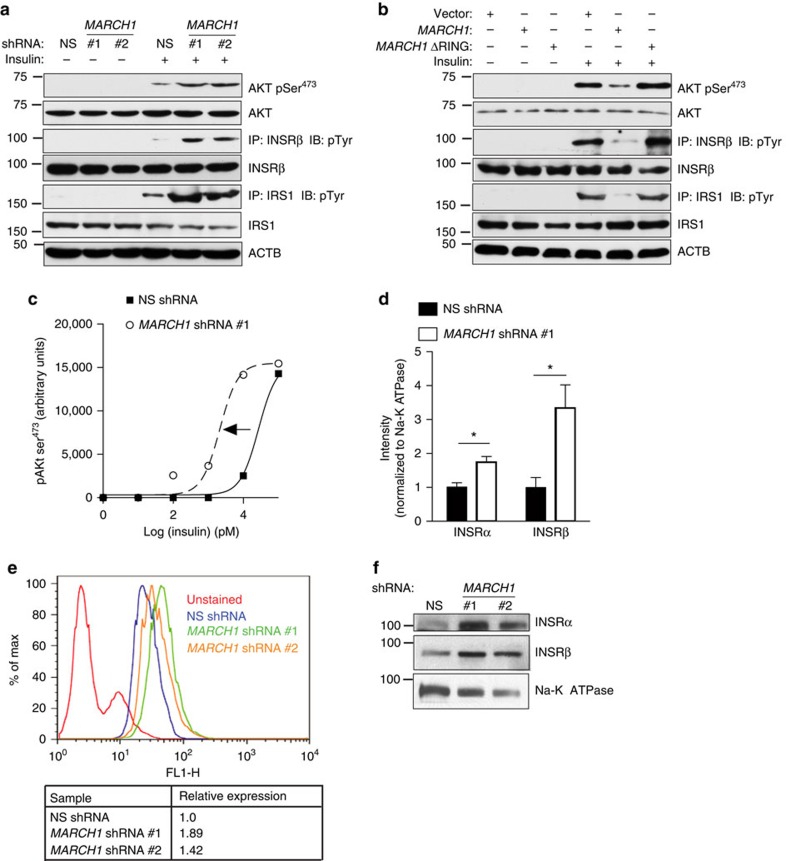
MARCH1 regulates insulin sensitivity by controlling surface INSR expression. (**a**) Immunoblot analysis of insulin-stimulated INSR, IRS-1 and AKT activation in HeLa cells expressing the indicated shRNAs. (**b**) Immunoblot analysis of insulin-stimulated INSR, IRS1 and AKT activation in HeLa cells expressing the indicated constructs. (**c**) Insulin dose–response curves in HeLa cells expressing NS or *MARCH1* shRNA #1, constructed using AKT pSer^473^ immunoblot densitometry (see also Supplementary Fig. 6d). (**d**) Biotinylation assay for cell surface expression of INSRα and INSRβ in serum-starved HeLa cells expressing NS or *MARCH1* shRNA #1, normalized to Na-K ATPase intensity (see also Supplementary Fig. 7a). (**e**) Flow cytometric measurement of surface INSRα in HeLa cells expressing the indicated shRNA. (**f**) Immunoblot analysis for cell surface expression of INSRα and INSRβ in GPMVs isolated from serum-starved HeLa cells expressing indicated shRNAs. Data are mean±s.e.m. In (**d**), *n=*3 biological replicates. In all panels, *P<0.05, comparisons by unpaired *t*-test.

After confirming a physiological and pathophysiological role for MARCH1 in insulin signalling, we next aimed to determine the mechanism by which MARCH1 impairs insulin action. *MARCH1* knockdown in HeLa and 3T3-L1 cells stimulated with suboptimal insulin was associated with enhanced AKT, IRS1 and INSR phosphorylation, indicating that the insulin-sensitizing effect of *MARCH1* knockdown could be traced to enhanced INSR activation (Fig. 7a and Supplementary Fig. 6a). Similarly, ectopic expression of wild-type MARCH1 was sufficient to inhibit INSR tyrosine phosphorylation, with consequent effects on IRS1 and AKT phosphorylation (Fig. 7b). The RING-CH E3 ligase domain of MARCH1 was required for this function; MARCH1 ΔRING failed to repress INSR, IRS1 and AKT phosphorylation (Fig. 7b). These results demonstrate that MARCH1 control of insulin signalling occurs by modulation of insulin receptor activation.

Because the effect of MARCH1 on insulin signalling was traceable to differential INSR tyrosine phosphorylation, we considered mechanisms that would account for differences at this step. In insulin time course experiments in HeLa and 3T3-L1 cells, *MARCH1* knockdown increased AKT Ser^473^ phosphorylation at early time points but was surprisingly associated with faster signal decay, suggesting that unlike other ubiquitin ligases implicated in insulin action, MARCH1 does not act by promoting ligand-induced internalization and degradation of INSR (Supplementary Fig. 6b,c). However, in insulin dose–response experiments, HeLa cells expressing *MARCH1* shRNA displayed a left-shifted dose–response function with no increase in maximal responsiveness—suggestive of increased surface INSR^9^ (Fig. 7c and Supplementary Fig. 6d–f). Since total cell lysate INSR content was unaltered by *MARCH1* knockdown (Fig. 7a and Supplementary Fig. 6a), we asked if MARCH1 specifically altered surface expression of INSR. We measured the surface INSR pool in HeLa cells expressing *MARCH1* shRNA using three independent techniques: biotinylation assays, flow cytometry and giant plasma membrane vesicle (GPMV) preparations. All three approaches revealed increased surface INSR levels in cells expressing *MARCH1* shRNA (Fig. 7d–f and Supplementary Fig. 7a,b). Other potential mechanisms for MARCH1 control of INSR tyrosine phosphorylation, including regulation of PTP1B, changes in dynamin-mediated INSR endocytosis and altered INSR compartmentation into lipid rafts/caveolae, were found not to be involved in the phenotype (Supplementary Fig. 7c–f).

### MARCH1 regulates INSR stability by direct ubiquitination

**Figure 8:**
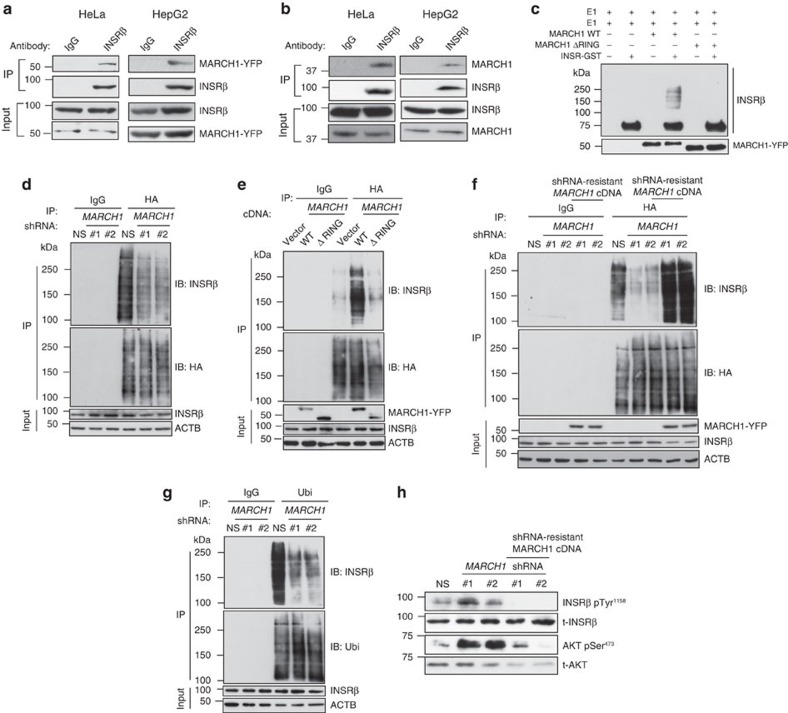
MARCH1 interacts with INSR and regulates its half-life by ubiquitination. (**a**) Co-immunoprecipitation of INSRβ and MARCH1 from HeLa and HepG2 cells expressing *MARCH1*-*YFP*. (**b**) Co-immunoprecipitation of INSRβ and endogenous MARCH1 from HeLa and HepG2 cells treated with the proteasome inhibitor MG132. (**c**) *In vitro* ubiquitination assay. Input was analysed for MARCH1 expression. (**d**) Immunoblot analysis of INSRβ ubiquitination. HA-ubiquitinated proteins immunoprecipitated from HeLa cells expressing NS or *MARCH1* shRNAs and HA-ubiquitin were immunoblotted for INSRβ and for HA-ubiquitin. Lysates were probed for the indicated proteins. (**e**) Immunoblot analysis of INSRβ polyubiquitination. HA-ubiquitinated proteins immunoprecipitated from HeLa cells expressing *MARCH1-YFP* or *MARCH1-ΔRING-YFP* and HA-ubiquitin were immunoblotted for INSRβ and for HA-ubiquitin. Lysates were probed for indicated proteins. (**f**) Immunoblot analysis of INSRβ polyubiquitination. HA-ubiquitinated proteins immunoprecipitated from HeLa cells expressing NS or *MARCH1* shRNA #1 or #2 with or without shRNA-resistant *MARCH1* cDNA were immunoblotted for INSRβ and HA-ubiquitin. Lysates were probed for indicated proteins. (**g**) Immunoblot analysis of INSRβ ubiquitination. Ubiqitinated proteins immunoprecipitated from HeLa cells expressing NS or *MARCH1* shRNAs were immunoblotted for INSRβ and for Ubiquitin. Lysates were probed for the indicated proteins. (**h**) Immunoblot analysis of insulin-stimulated INSR and AKT activation in HeLa cells. HeLa cells expressing NS or *MARCH1* shRNAs with or without shRNA-resistant *MARCH1* cDNA were analysed for indicated proteins.

We next examined whether MARCH1 control of the surface INSR pool was mediated by direct ubiquitination. Co-immunoprecipitation experiments in HeLa and HepG2 cells expressing epitope-tagged constructs revealed that MARCH1 and INSRβ interact (Fig. 8a). We were also able to detect this interaction in endogenous proteins from both HeLa and HepG2 cells, but only when MARCH1 levels were stabilized using the proteasome inhibitor MG132 (Fig. 8b). Without proteasome inhibition, MARCH1 autoubiquitination keeps its protein levels below the limit of detection of immunoblotting^23^,^26^,^27^,^28^. We also generated several *MARCH1* deletion mutants to map the MARCH1 domains required for interaction with INSRβ. These experiments revealed that both transmembrane domains as well as an N-terminal cytoplasmic domain, but not the RING E3 ligase domain or the C-terminal domain, are required for the MARCH1-INSRβ interaction (Supplementary Fig. 8a,b). Also, deletion of either of the transmembrane domains partially inhibited the localization of MARCH1 to the plasma membrane, compared with the wild-type MARCH1 (Supplementary Fig. 8c). Furthermore, immunoprecipitated wild-type MARCH1, but not E3-ligase activity-defective MARCH1, polyubiquitinated the INSRβ kinase domain *in vitro* (Fig. 8c). To examine whether MARCH1 ubiquitinates INSRβ in intact cells, we measured INSRβ ubiquitination while modulating MARCH1 levels. We found that *MARCH1* knockdown decreased INSRβ ubiquitination (Fig. 8d and Supplementary Fig. 9a), and that ectopic expression of wild-type, but not E3 ligase defective, *MARCH1* was associated with increased INSRβ polyubiquitination in cells (Fig. 8e and Supplementary Fig. 9b). To confirm that these effects are directly mediated by MARCH1, we performed rescue experiments measuring INSRβ ubiquitination in *MARCH1* shRNA-treated cells in the presence or absence of an shRNA-resistant *MARCH1* cDNA. *MARCH1* shRNA reduced INSRβ ubiquitination relative to controls, but ectopic expression of this construct rescued this decrease in ubiquitination (Fig. 8f and Supplementary Fig. 9c). To rule out potential confounding effects of ectopic HA-ubiquitin expression, we also measured endogenous polyubiquitination of INSRβ and consistent with our other results, observed that *MARCH1* knockdown reduced INSRβ polyubiquitination (Fig. 8g). Furthermore, consistent with these results, *MARCH1* shRNA increased insulin-stimulated INSR and AKT phosphorylation, but this effect was reversed upon expression of shRNA-resistant *MARCH1* cDNA (Fig. 8h).

**Figure 9:**
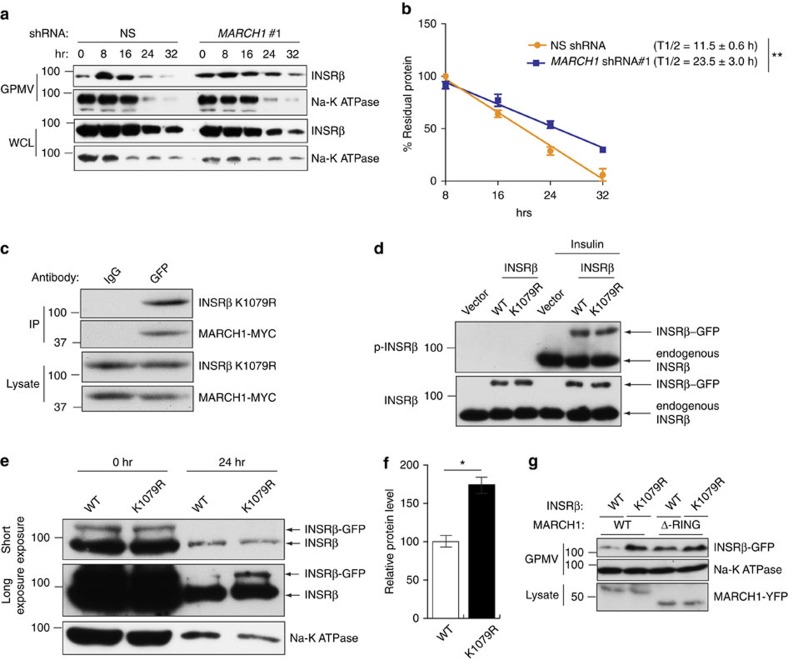
MARCH1 ubiquitination of INSRβ Lys^1079^ controls INSRβ membrane stability. (**a**) Surface INSRβ content in GPMVs from HeLa cells expressing the indicated shRNAs and treated with cycloheximide (see also Supplementary Fig. 7a). (**b**) Analysis of surface INSRβ half-life, normalized to Na-K ATPase intensity (*n*=3). (**c**) Co-immunoprecipitation of INSRβ K1079R-GFP and MARCH1-MYC from HeLa cells expressing both proteins. (**d**) Immunoblot analysis of INSRβ phosphorylation in HeLa cells expressing vector, INSR-GFP WT or INSR K1079R-GFP with or without insulin stimulation. (**e**) Immunoblot analysis of INSRβ and Na-K ATPase surface stability in GPMVs of HeLa cells expressing wild-type or K1079R INSR and treated with cycloheximide. Short (top) and long (middle) exposures of INSRβ blots are shown. (**f**) Densitometric quantitation (*n*=3) of bands labelled as INSRβ-GFP in **e**. (**g**) HeLa cells were transfected with wild-type or K1079R mutant INSR-GFP and either wild-type or ΔRING MARCH1-YFP constructs. GPMVs were analysed for INSRβ and Na-K ATPase and lysates were analysed for MARCH1-YFP by immmunoblot analysis. Data are mean±s.e.m. In (**e**), *n=*3 biological replicates. In all panels, *P<0.05, comparisons by *t*-test.

We anticipated that ubiquitination of INSRβ by MARCH1 would increase INSRβ turnover. Consistent with this hypothesis, *MARCH1* knockdown was associated with increased surface INSRβ half-life in serum-starved cells (Fig. 9a,b and Supplementary Fig. 10a,b). In the presence of insulin, this effect on half-life was lost—likely reflecting action of insulin-stimulated INSR ubiquitination pathways (Supplementary Fig. 10c–f). To further examine this potential contrast with established INSR ubiquitination pathways, such as the feedback inhibition of CBL and NEDD4, we used GPMV preparations to measure surface INSRβ expression in cells expressing shRNA for either *MARCH1*, *CBL* or *NEDD4*. Cells with knockdown of *CBL* or *NEDD4* displayed increased surface INSR after insulin stimulation, but cells with knockdown of *MARCH1* only displayed increased surface INSR in the basal state (Supplementary Fig. 10g,h). These data support the hypothesis that MARCH1 regulates surface INSR levels specifically in the basal, low-insulin state—establishing a new paradigm for E3 ligase–INSR interactions.

To determine the MARCH1 ubiquitination site on INSRβ, we performed LC-MS/MS analysis of immunoprecipitated INSRβ. These experiments identified INSR Lys^1079^ as preferentially ubiquitinated in cells overexpressing MARCH1 compared with empty vector control (Supplementary Fig. 11). To test the role of MARCH1-mediated ubiquitination of INSRβ at Lys^1079^, we mutated Lys^1079^ to arginine (K1079R). INSRβ K1079R mutation did not affect the INSRβ-MARCH1 interaction or insulin-stimulated INSR autophosphorylation (Fig. 9c,d). However, INSRβ K1079R displayed increased cell surface stability (Fig. 9e–g). Collectively, these results identify INSRβ Lys^1079^ as a potential MARCH1 substrate capable of regulating surface INSRβ expression.

## Discussion

**Figure 10:**
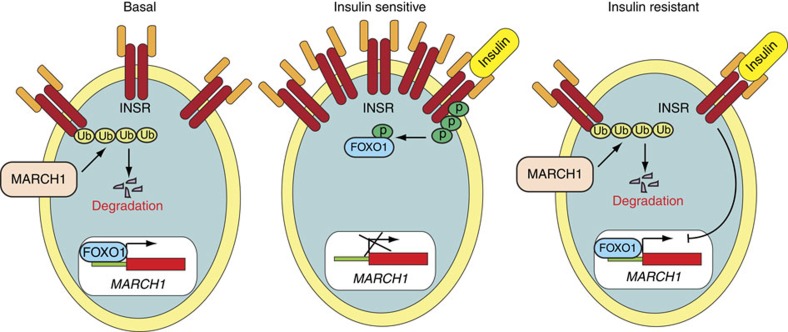
Model for MARCH1-mediated regulation of insulin action. In the basal state, MARCH1 ubiquitinates and degrades INSRβ; thereby reducing surface INSR expression. In the insulin-sensitive state, INSR activation inhibits FOXO1, resulting in transcriptional repression of *MARCH1*, increased surface INSR levels, and preserved insulin signalling. In the insulin-resistant state, insulin fails to inhibit FOXO1, resulting in inappropriately increased MARCH1 expression, decreased surface INSR levels, and impaired insulin signalling.

Our results, summarized in Fig. 10, identify the E3 ubiquitin ligase MARCH1 as a new repressor of insulin receptor signalling. We found that MARCH1 potently inhibits cellular insulin sensitivity in multiple cell types including hepatocytes and white adipocytes, and is itself transcriptionally regulated by insulin through the transcription factor FOXO1. *In vivo* knockdown of *March1* expression in liver and WAT enhanced insulin sensitivity in regular chow-fed mice and prevented insulin resistance in high-fat-fed mice. Conversely, AAV-induced ectopic expression of *March1* in mouse liver was sufficient to induce insulin resistance in regular chow-fed mice. The effect of MARCH1 on cellular insulin sensitivity was mediated through direct ubiquitination of INSRβ by MARCH1. By altering the half-life of surface INSRβ, MARCH1 controls the surface INSR pool and thus regulates insulin sensitivity.

Ubiquitination plays a central role in many biological processes^29^. The ubiquitin pathway involves the sequential transfer of ubiquitin from an ubiquitin-activating enzyme (E1) to a ubiquitin conjugase (E2) and then to a substrate protein via the action of an E3 ubiquitin ligase. The human genome encodes several hundred E3 ligases and their adapter proteins, which provide specificity to the ubiquitin pathway^30^. The MARCH family of E3 ubiquitin ligases contains eleven genes, most of which contain at least two transmembrane regions^31^,^32^. MARCH family proteins are known to downregulate a subset of membrane proteins, but determining specific physiological roles for MARCH isoforms has proven difficult owing in part to limited understanding of their expression, regulation and substrates^32^,^33^. MARCH1 is known to ubiquitinate MHC class II in immature dendritic cells, promoting degradation and preventing trafficking to the plasma membrane^28^. However, a role for MARCH1 in the regulation of metabolism or insulin signalling has not to our knowledge been described.

Two other ubiquitin ligases were identified and validated as repressors of insulin signalling by our RNAi screen: *MARCH9* and *NHLRC1*. Because *MARCH1*, unlike *MARCH9* and *NHLRC1*, displayed upregulation in obesity, it was the focus of the present study. However, *MARCH9* and *NHLRC1* may also regulate cellular insulin action, perhaps through direct ubiquitination of INSR. Although *MARCH8*, not *MARCH9*, is the *MARCH* family member most closely related to *MARCH1* (ref. 31), we cannot rule out the possibility that *MARCH9* (or *NHLRC1*) acts through a similar mechanism as *MARCH1*.

Other ubiquitin ligases have been shown to ubiquitinate the insulin receptor. Most prominent among these is CBL, which is recruited to the activated INSR (and to other activated receptor tyrosine kinases), marking it for internalization and degradation^18^,^21^. The ubiquitin ligase NEDD4 also participates in a ligand-induced repression of insulin signalling by ubiquitinating INSR through the adapter protein GRB10^19^,^21^,^34^,^35^. The RING E3 ligase MG53 has been suggested to ubiquitinate INSR in skeletal muscle, though this is controversial^17^,^19^.

However, surprisingly, we find that the mechanism by which MARCH1 regulates INSR activity differs significantly from these established paradigms. Whereas CBL and NEDD4 function in negative feedback loops that are activated after insulin stimulation, MARCH1 appears to act in the basal state to tune cellular insulin sensitivity. In our studies of cells expressing *MARCH1* shRNA, surface INSR content was increased most prominently in the basal, serum-starved state. Indeed, our measurements of surface INSRβ half-life in cells expressing *MARCH1* shRNA revealed differences only in the basal state, not during insulin stimulation. These data are consistent with a model in which MARCH1 regulates the constitutive process of receptor turnover rather than the insulin-stimulated process of internalization and degradation of phosphorylated receptors. This interpretation is further substantiated by previous studies demonstrating that MARCH1 can alter the fate of endocytosed membrane proteins to favour degradation over recycling^36^.

MARCH1 regulation of INSR signalling is unlike regulation by CBL and NEDD4 in another significant way: it appears to function in a positive feedback loop. Specifically, we observed that insulin acutely decreases *MARCH1* expression through a canonical FOXO1-mediated mechanism. This would tend to promote increased surface INSR content after insulin stimulation, though with the requisite time delay associated with transcriptionally mediated processes. Such a delayed mechanism could serve an important physiological role by counteracting the acute ligand-stimulated internalization and degradation of activated INSR (itself a ubiquitin-mediated process through CBL and NEDD4). In this way, insulin regulation of MARCH1 could contribute to the resetting of cellular insulin sensitivity in the postprandial state. The dysregulation of FOXO1 that accompanies insulin resistance is reflected in our observation that MARCH1 expression is increased in WAT from obese adolescent humans. Such an effect would exacerbate insulin resistance and is consistent with the well-established decreased surface INSR content in adipocytes from obese humans^37^,^38^. However, because FOXO1 dysregulation must precede dysregulation of insulin control of MARCH1, this phenomenon is likely to be consequence rather than cause of insulin resistance.

Our observation that plasma membrane INSR content is increased in cells expressing *MARCH1* shRNA is congruent with our insulin dose–response data. Although the plasma membranes of insulin-responsive cells have long been appreciated to contain ‘spare receptors' such that a maximal insulin signalling response can be achieved with only a small fraction of receptors occupied, alterations in surface INSR content are well established to affect insulin signalling at submaximal insulin concentrations^9^,^39^,^40^,^41^. This prediction matches our experimental data, which show increased INSR signalling at submaximal insulin doses (in effect, decreasing the physiological ED_50_ for insulin action) without any increase in maximal insulin responsiveness at higher insulin doses. It is interesting to note that this left shift, despite not increasing maximal insulin responsiveness, significantly improved glucose tolerance and insulin sensitivity in mice treated with the *March1* ASO and in *March1*^*−/−*^ mice. These results, especially considering the modest effectiveness of the *March1* ASO (60 and 80% knockdown in liver and WAT, respectively), suggests that enhanced insulin sensitivity even only at suboptimal insulin levels can cause significant improvements in whole-body metabolism.

Our study also has some limitations. A major challenge for investigations of MARCH1 biology is its extraordinarily low protein expression, thought to be a consequence of autoubiquitination^23^. As a result, our data linking MARCH1 levels to metabolic phenotypes are based on mRNA expression rather than the more relevant protein expression. This phenomenon also prevented interrogation of the MARCH1-INSR interaction in mouse liver. In addition, although we observed significant *MARCH1* upregulation in WAT from obese insulin-resistant human adolescents, the quantitative contribution of this upregulation to cellular insulin action is uncertain. Another caveat is that because our study focused on classical insulin target tissues such as liver and WAT, the role of other cell types with high *MARCH1* expression (for example, antigen-presenting cells) was not examined in our mouse metabolic phenotyping studies. Finally, all mechanistic studies presented in this study were performed in cultured cells. Further work is needed to determine if these mechanisms, particularly MARCH1 ubiquitination of INSRβ, are operative *in vivo*.

Our results have broad significance for understanding insulin action and resistance in health and disease. First, the mechanism by which MARCH1 regulates INSR represents a new paradigm for E3 ligase—INSR interactions. Rather than functioning in an insulin-activated negative feedback loop, MARCH1 appears to act in the basal state to control the gain of insulin action. Second, analysis of mouse and human WAT revealed that MARCH1 expression is inappropriately increased in obesity. This, paired with our observation that insulin normally represses MARCH1 expression, proposes a mechanism for the long-observed but incompletely understood phenomenon of surface INSR downregulation in insulin resistance^9^,^40^,^41^: de-repression of MARCH1. Taken together, our data highlight the utility of unbiased, large-scale screens to uncover novel regulators of cellular pathways and suggest that despite intense interest in targeting downstream effectors of insulin action, the insulin receptor itself may be a tunable locus of control for insulin resistance and T2D.

## Methods

### RNA interference screen

HeLa cells were transduced with three lentiviral shRNA pools containing 2,833 shRNAs against 616 E3 ligase genes or NS shRNA in triplicate at 0.2 multiplicity of infection (MOI) to prevent superinfection and to ensure that each cell received no more than one shRNA. After infection, HeLa cells were selected with puromycin (0.2 μg ml^−1^) for 7 days to enrich for HeLa cells expressing shRNA. After puromycin selection, HeLa cells were grown in serum-free DMEM containing trace elements (D0547, Sigma) supplemented with 50 ng ml^−1^ EGF (E9644, Sigma), 20 ng ml^−1^ FGF (SRP3043, Sigma), 100 nM hydrocortisone (H0888, Sigma), 0.5 μg ml^−1^ transferrin (T1147, Sigma), 5 ng ml^−1^ selenium (S5261, Sigma), 0.5 μg ml^−1^ fibronectin (F1141, Sigma) and 0.05 μg ml^−1^ insulin (I0516, Sigma). Media was changed every 3 days and cells were split at a 1:4 ratio every 7 days. After four passages all cells carrying NS and shRNAs from pool 2 were dead. Surviving cells from pools 1 and 3 were collected and genomic DNA was isolated. The integrated shRNAs were PCR-amplified using primers specific to the shRNA vector (pLKO.1) and listed in Supplementary Table 3. Samples were sequenced using primer SP6 (Supplementary Table 3) to identify candidate shRNAs.

### Cell culture, plasmids and cloning

Authenticated ATCC cell lines HepG2 and HeLa were purchased from ATCC (HepG2, ATCC # HB-8065 and HeLa, ATCC # CCL-2). HepG2 and HeLa were grown in DMEM containing 10% FBS at 37 °C at % CO2. 3T3-L1 cells (ATCC # CL-173) were a kind gift from Dr. Jonathan Bogan (Yale University). 3T3-L1 cells were maintained in DMEM containing 10% calf serum. All cell lines were tested for mycoplasma contamination by ATCC using Hoechst DNA staining method, agar culture and a PCR-based assay. All cell lines were verified by ATCC using short tandem repeat profiling analysis. Differentiated 3T3-L1 adipocytes were maintained in DMEM with 10% FBS and 1 μg ml^−1^ insulin for 5-11 days before study. The plasmids pCDNA3.1 MARCH1-YFP, MARCH1ΔRING-YFP, MARCH1ΔCyto-YFP, MARCH1ΔC-Term-YFP, pCDNA3.1 MARCH1-Myc were as described^37^,^23^. hIR-GFP was obtained from Joseph Bass (Addgene #22286). The human MARCH1 promoter (2.3 kb upstream of the transcription start site) was PCR-amplified and cloned into plasmid pGL4.14 (GE Lifesciences) between KpnI and XhoI. pcDNA GFP FKHR and pcDNA GFP FKHR AAA (constitutively active) were obtained from William Sellers (Addgene #9022, #9023). pcDNA4 myc PGC-1α was obtained from Toren Finkel (Addgene #10974).

### Site-directed mutagenesis

hIR K1079R-GFP was generated through site-directed mutagenesis of hIR-GFP using specific primers (Supplementary Table 3). The FOXO1-binding site ‘ GTAAACA ' (-1,343 to -1,337 transcription start site) was mutated to ‘ GTACCA ' using specific primers (Supplementary Table 3). Specific deletions of the first and second transmembrane domains of MARCH1 were achieved with specific deletion primers. shRNA-resistant MARCH1 cDNAs were generated using shRNA#1 or shRNA#2-specific primers by mutating the third base of the amino acid codons without altering the amino acid composition at the shRNA-targeting sites. All site-directed mutagenesis reactions were performed using Quikchange II (Agilent) as per the manufacturer's protocol.

### RNA purification, cDNA synthesis and qPCR

Total mRNA was prepared using TRIzol (Life Technologies) and purified using RNeasy mini columns (Qiagen). For mRNA expression analyses, cDNA was generated using the M-MuLV First Strand cDNA Synthesis kit (New England Biolabs). Quantitative real-time PCR was performed using Power SYBR Green kit (Applied Biosystems). Primers used for analysing mRNA expression are listed in Supplementary Table 3.

### shRNAs and lentivirus preparation

pLKO.1 lentiviral vector-based shRNAs against candidate genes and non-silencing shRNA were obtained from OpenBiosystems. shRNA and siRNA information are provided in Supplementary Table 4. Lentivirus particles were prepared using 293 T cells by transfecting either gene-specific shRNA or non-silencing shRNA plasmids along with the lentiviral packaging plasmids as described ( https://www.broadinstitute.org/rnai/trc/lib). All lentiviral transfections were performed using Effectene (Qiagen). Stable cell lines were generated by infection with lentivirus particles and selected with puromycin to enrich for infected cells.

### Antibodies and immunoblot analysis

Whole-cell protein extracts were prepared using IP lysis buffer (Pierce) containing protease inhibitor cocktail (Roche) and phosphatase inhibitor cocktail (Sigma). Protein concentration was estimated using the Bradford assay (Bio-Rad). Proteins were separated onto 10 or 12% polyacrylamide gels and transferred onto polyvinylidene difluoride membrane by wet transfer. Polyvinylidene difluoride membranes were blocked with 5% nonfat dry milk or 5% BSA, washed, probed with primary antibodies, washed, and incubated with horseradish peroxidase-conjugated secondary antibodies (GE Healthcare). Immunoblots were developed using Supersignal Pico or Femto reagent (Pierce), as needed. Primary antibodies used are listed in Supplementary Table 3. Uncropped images of all western blots are provided along with size markers in Supplementary Fig. 12. In case of multiple bands the relevant bands are boxed in red.

### Luciferase reporter assay

HeLa cells were transfected with wild-type MARCH1 reporter or FOXO1-binding site mutant MARCH1 reporter; either empty vector, pcDNA GFP FKHR or pcDNA GFP FKHR AAA (constitutively active); and either empty vector or pcDNA4/MYC-PGC-1α as indicated. Twenty-four hours after transfection, cells were serum starved overnight. Cells were stimulated with insulin (5 μg ml^−1^) or left untreated for 2 h, then lysed. The luciferase reporter assay was performed using a dual luciferase assay kit (Promega). pRL-TK was used as transfection control. Relative reporter activity was measured as ratio of firefly to renilla luciferase activity after setting vector co-transfected and non-insulin stimulated cells to 1.

### ChIP assays

ChIP was performed as described previously^42^. Briefly, HeLa cells that were either insulin-stimulated (5 μg ml^−1^) or untreated for 2 h following serum starvation were fixed with 1% formaldehyde and lysed in SDS lysis buffer (1% SDS, 50 mM Tris-HCl (pH 8.0), 10 mM EDTA and protease inhibitor cocktail (Roche)). Following lysis, chromatin was sheared by sonication and immunoprecipitated with FOXO1 antibody (Supplementary Table 3). Chromatin was eluted after low-salt, high-salt and LiCl_2_ wash. DNA was precipitated after reversal of crosslinking at 65 °C. Quantitative PCR was performed using primers listed in Supplementary Table 3. Fold enrichment was calculated as the ratio of immunoprecipitated DNA to input DNA.

### *In vitro* ubiquitination assay

The *in vitro* ubiquitination assay was performed using a ubiquitin conjugation initiation kit (Boston Biochem). MARCH1-YFP or MARCH1ΔRING-YFP immunoprecipitated from HeLa cells was used as E3. The reaction was carried out in a final volume of 20 μl, to which 10 μl of IgG beads were added. 500 ng of GST-tagged insulin receptor kinase domain, residues 941-1343 (Enzo) was used as substrate. One microgram of purified E2 enzyme UBE2R1 (Ubiquigent) was used in each reaction. Ubiquitination reaction products were resolved by 6% PAGE and immunoblotted for INSRβ.

### *In vivo* ubiquitination assay

HeLa cells expressing NS or *MARCH1* shRNAs were transfected with HA-ubiquitin expression vectors or left untransfected and serum starved. MG132 (10 μM) was added 4 h before lysis. Cells were lysed in IP lysis buffer (Pierce) containing 100 mM *N*-ethylmaleimide (NEM). Immunoprecipitation was performed with isotype control IgG or Anti-HA or Anti-Ubi (FK2) antibodies and the product were analysed for ubiquitinated INSRβ by immunoblotting. The immunoprecipitate was also analysed for total ubiquitinated or HA-ubiquitinated proteins, depending on the antibody used for ubiquitin immunoprecipitation. To measure rescue of INSR ubiquitination with shRNA-resistant *MARCH1* cDNA, HeLa cells expressing NS or *MARCH1* shRNA were transfected with INSR-GFP and HA-ubiquitin expression vectors and empty vector or corresponding shRNA-resistant *MARCH1* cDNA. Immunoprecipitation was performed with IgG or anti-HA antibodies and the product was analysed for ubiquitinated INSRβ by immunoblotting with anti-GFP antibody. For analysis of INSRβ ubiquitination with *MARCH1* overexpression, HeLa cells were transfected with INSR-GFP, HA-Ubiquitin and either empty vector or *MARCH1*-Myc WT or *MARCH1*ΔRING-Myc and the samples were processed as above. Reciprocally, ubiquitination assays were also performed by immunoprecipitation with anti-INSRβ or anti-GFP antibody as indicated followed by immunoblotting with anti-HA antibody. The INSRβ or INSRβ-GFP immunoprecipitates treated with USP2 enzyme were analysed for INSRβ or INSRβ-GFP (ref. 43).

### Liquid chromatography/mass spectrometry (LC/MS) analysis

HeLa cells were transfected with empty vector, MARCH1-Myc, or MARCH1ΔRING-Myc along with INSR-GFP and HA-Ubiquitin. Twenty-hour after transfection, cells were serum starved overnight. MG132 was added 4 h before lysis in Co-IP Buffer (Pierce). INSR-GFP was immunoprecipitated using anti-GFP antibody and eluted in 200 μl 0.2 M glycine, pH 2.5. The eluate was partially dried to ∼100 μl and precipitated with MeOH:CHCl_3_:Water (4:1:3 ratio). The pellet was dried and reconstituted in 8 M urea, 0.4 M ammonium bicarbonate, reduced with DTT, and alkylated with iodoacetamide in the dark. The sample was then trypsin-digested overnight at 37 °C. The digested sample was injected onto a Q-Exactive Plus (Thermo Fisher) liquid chromatography–tandem mass spectrometry system equipped with a Waters Symmetry C18 180 μm × 20 mm trap column and a 1.7 μm, 75 μm × 250 mm nanoACQUITY UPLC column (37 °C) for peptide separation. Trapping was performed at 5 μl min^−1^, 99% Buffer A (100% water, 0.1% formic acid) for 3 min. Peptide separation was performed with a linear gradient over 140 min at a flow rate of 300 nl min^−1^. Collected data were processed using MASCOT Distiller and Search Engine (v.2.4), and modification sites were manually verified.

### Flow cytometry analysis

Fluorescence-activated cell sorting analysis of cell surface INSR was performed using antibody against INSRα (Pierce) as per manufacturer's protocol. Briefly, overnight serum starved HeLa cells carrying either NS or MARCH1 shRNA left untreated were fixed with 2% paraformaldehyde followed by blocking with 1% BSA and primary antibody staining at 1:100 dilution (Supplementary Table 3). Alexa 488-conjugated IgG (Invitrogen) was used as secondary antibody at 1:200 dilution and staining intensity was measured using a FACSCalibur Analyzer (BD Biosciences).

### Biotinylation and giant plasma membrane vesicle assays

Biotinylation assays were performed on overnight serum starved cells using the Cell Surface Protein Isolation Kit (Pierce) according to the manufacturer's instructions. For biotinylation assays to measure insulin induced INSR membrane trafficking, HeLa cells carrying NS or shRNAs against MARCH1 or CBL or NEDD4 were used. Cells were overnight serum starved and either left untreated or insulin treated for 3 h at an insulin concentration of 5 μg ml^−1^ insulin.

GPMVs were isolated as described previously^44^. GPMVs were lysed in Laemmli SDS–polyacrylamide gel electrophoresis buffer and analysed by immunoblotting for INSRα, INSRβ and Na-K ATPase. To measure the effect of Dynasore on plasma membrane INSR expression, HeLa cells carrying indicated shRNAs were serum starved overnight and were treated with DMSO or 20 μM Dynasore. One of these groups were stimulated for 3 h with 5 μg ml^−1^ insulin.

For half-life estimation of cell surface INSR, HeLa cells were seeded as above and after overnight serum starvation 100 μg ml^−1^ of cycloheximide was added with or without 5 μg ml^−1^ insulin. GPMVs were collected at indicated time points. In parallel, whole-cell lysates were collected at the indicated time points. GPMVs and lysate were analysed for INSRβ and Na-K ATPase levels. The densitometry values of cell surface INSRβ normalized to Na-K ATPase were graphed and half-life was calculated for periods of linear steady state degradation from 8 to 32 h for insulin-free and 0 to 16 h for insulin-treated conditions.

### Co-immunoprecipitation assay

HeLa or HepG2 cells either untransfected or transfected with MARCH1 or MARCH1 deletion constructs were treated with 5 μM MG132 overnight. Cells were lysed in IP Lysis buffer (Pierce) and lysates were used for immunoprecipitation with IgG or anti-INSRβ antibody and protein G-agarose beads (Invitrogen). For co-immunoprecipitation of mutant INSRβ and MARCH1, HeLa cells were transfected with INSR-GFP or INSR K1079R-GFP along with MARCH1-Myc. INSRβ-GFP was immunoprecipitated with either IgG or anti-GFP antibody and analysed by immunoblot for indicated proteins. To avoid interference of antibody heavy chain in immunoblot wherever possible, antibodies for immunoprecipitation and immunoblot raised in different species were chosen. Primary antibodies used are listed in Supplementary Table 3.

### Glycogen synthesis assay

A total of 5 × 10^5^ HeLa, differentiated 3T3-L1 adipocytes or HepG2 cells were serum starved overnight and treated with 0.05 μg ml^−1^ insulin for 30 min or left untreated. Cells were washed twice in ice-cold PBS, scraped in 100 μl of water and boiled for 5 min. To 25 μl of cell lysate, 25 μl of 0.1 M sodium acetate containing 0.4 mg ml^−1^ amyloglucosidase was added. The reaction was incubated at 37 °C for 90 min. One hundred microlitre of glucose oxidase reaction mix (Sigma) was added and reactions were incubated for 30 min at 37 °C. The reaction was stopped by adding 100 μl 12*N* H_2_SO_4_ and absorbance was measured at 540 nm. Total glycogen was calculated using a standard curve of glycogen. Relative glycogen synthesis was calculated by normalizing to non-insulin stimulated cells carrying NS shRNA.

### Glucose uptake assay

HeLa cells, HepG2 cells or differentiated 3T3-L1 adipocytes in 12-well plates were incubated in low glucose media (5 mM glucose) for 2.5 h followed by glucose-free media with or without 100 nM insulin for 30 min. 0.2 μCi of [1-^14^C]-2-deoxyglucose (2-DG) and 200 μM nonradioactive 2-DG were added to each well and incubated for 2.5–5 min at room temperature. The reaction was terminated by adding 2-DG to 200 mM. The plates were washed thrice in ice-cold PBS and cells were scraped in 500 μl deionized water. ^14^C radioactivity was assessed by scintillation counting. Relative glucose uptake rate was calculated by setting non-insulin stimulated cells carrying NS shRNA as 1.

### Sucrose gradient fractionation

Subcellular fractions enriched for lipid rafts and caveolae were prepared by discontinuous 5–45% sucrose gradient ultracentrifugation as described previously^45^. Fractions collected from the top of the gradient were boiled in Laemmli SDS–polyacrylamide gel electrophoresis sample buffer and analysed for INSR and marker protein content by immunoblotting.

### Animals

All experimental procedures were approved by the Institutional Animal Care and Use Committee (IACUC) of Yale University School of Medicine before study initiation. Male 12–16-week-old C57BL/6 J mice were obtained from the Jackson Laboratory for ASO and AAV studies and studied at 14-20 weeks of age. *March*^*−/−*^mice were previously generated^27^ on the C57BL/6 background and were maintained with heterozygote breeders so that littermate wild-type controls could be used for metabolic studies. *March1*^*−/−*^ mice were studied at 12–20 weeks of age. Mice received free access to food and water and were housed with 12 h light/dark cycles (0700–1900 hours) at 23 °C. Diets used were: regular chow (Harlan Teklad TD2018S, 18% fat, 58% carbohydrate, 24% protein); HFD (Research Diets D12492, 60% fat, 20% carbohydrate, 20% protein). For studies of candidate gene expression in obesity, mice were fed HFD for 4 weeks. For all animal studies, sample sizes were selected to yield 90% power (at *α*=0.05) to detect 20% differences in metabolic parameters with an expected s.d. of 10%. Mice were randomly allocated to experimental groups, and weight-matching was ensured before beginning experimental protocols. Investigators were not blinded to treatment group during studies.

### Second-generation antisense oligonucleotide treatment

2'-*O*-methoxyethyl chimeric ASOs targeting *MARCH1* were synthesized as described^46^ and screened in mouse hepatocytes for target knockdown. Three candidate sequences were tested in mice. The most potent and nontoxic candidate was ISIS 671547, with the sequence 5′- GGCTCTGCTAACCAATATTC -3′. This ASO was used in all subsequent studies. The control ASO, ISIS 141923, has the sequence 5′- CCTTCCCTGAAGGTTCCTCC -3′ and is not complementary to any known mouse gene. ASO solutions in phosphate buffered saline were injected intraperitoneally biweekly at a dose of 75 mg kg^−1^ wk^−1^ for 2 weeks.

### Adeno-associated virus (AAV) treatment

Mouse *MARCH1* cDNA, isoform 3 (Origene) was cloned into the pscAAV2CB6 vector plasmid. AAV was produced as described previously using standard 293 cell triple transfection and CsCl gradient purification techniques^47^. Control AAV was scAAV8.CB6.eGFP. AAV was delivered by tail vein injection at 7.5 × 10^11^ genome copies per mouse. Mice were studied 4 weeks after AAV treatment to achieve maximal expression.

### Intraperitoneal glucose tolerance tests

Awake mice fasted 8 h (for *March1* ASO studies in male mice) or overnight (for *March1*^*−/−*^ studies in female mice) were placed under gentle tail restraint and injected intraperitoneally with 10% glucose solution at 1 mg g^−1^ (for *March1* ASO studies in male mice) or 2 mg g^−1^ (for *March1*^*−/−*^ studies in female mice). For the next 120 min, plasma was collected at regular intervals by tail massage for determination of plasma glucose and insulin concentrations.

### Hyperinsulinemic-euglycemic clamp studies

Jugular venous catheters were placed 6–9 days before study. Clamp studies and calculations were performed as previously described^48^ and in compliance with the standard operating procedures of the NIH Mouse Metabolic Phenotyping Centers (MMPC)^49^. Only mice that recovered to >90% of their preoperative body weight were studied. After fasting (6 h for *March1* ASO and *March1* AAV studies; 12 h for *March1*^*−/−*^ studies), awake mice under gentle tail restraint received a 120 min infusion of [3-^3^H]-glucose (0.05 μCi min^−1^) to measure basal glucose turnover. The hyperinsulinemic-euglycemic clamp lasted 140 min and was started with a 14.3 mU kg^−1^ prime bolus of insulin (Novolin, Novo Nordisk) delivered over 3 min followed by a continuous insulin infusion of 2 mU kg^−1^ min^−1^ (for *March1* ASO and chow-fed *March1*^*−/−*^ studies) or 21.5 mU kg^−1^ prime/3 mU kg^−1^ min^−1^ continuous (for *March1* AAV and HFD-fed *March1*^*−/−*^ studies). 20% glucose was infused at a variable rate to maintain euglycemia (100–120 mg dl^−1^) with [3-^3^H]-glucose added at a target infusion rate of 0.1 μCi min^−1^ (hot-GINF) to measure insulin-stimulated glucose turnover. Plasma was collected by tail bleeding for determination of plasma glucose, insulin, NEFAs and ^3^H-glucose specific activity. At the completion of the clamp study, mice were anaesthetized with pentobarbital sodium (150 mg kg^−1^). Tissues were rapidly harvested and snap-frozen in liquid N_2_ with pre-cooled clamps.

### Plasma measurements

Plasma glucose was measured using a YSI Biochemistry Analyzer (Yellow Springs Instruments). Plasma insulin was measured by radioimmunoassay (Linco). Plasma NEFAs were measured enzymatically (NEFA-HR, Wako). Plasma transaminase activity was measured by COBAS.

### Human adipose qPCR

WAT biopsies from lean and obese human adolescents were collected with written informed consent and Yale University Human Investigation Committee approval as part of the Yale Pathophysiology of T2D in Obese Youth Study^50^. Patients were stratified into lean (BMI<25) and obese (BMI>30) groups. A total of 1–2 g abdominal subcutaneous adipose tissue biopsies were collected inferior to the umbilicus after local lidocaine anaesthesia. RNA was isolated using the Qiagen Lipid Tissue Kit and cDNA synthesized using the M-MuLV First Strand cDNA Synthesis kit (New England Biolabs). Quantitative real-time PCR was performed using an Applied Biosystems 7500 Fast system.

### Statistical analysis

All values are expressed as the mean±s.e.m. The significance between the mean values for each study was evaluated by two-tailed unpaired Student's *t*-test (for comparisons of two groups) or analysis of variance (for comparisons of more than two groups) with the Holm–Sidak correction for multiple comparisons. Variance was calculated to be similar between groups compared.

### Data availability

All relevant data are available from the authors on request and/or are included with the manuscript (as figure source data or Supplementary Information files).

## Additional information

**How to cite this article:** Nagarajan, A. *et al*. MARCH1 regulates insulin sensitivity by controlling cell surface insulin receptor levels. *Nat. Commun.* 7:12639 doi: 10.1038/ncomms12639 (2016).

## Supplementary Material

Supplementary Information Supplementary Figures 1-12 and Supplementary Tables 1-4
