# Supplementary material for: MARCH1 regulates insulin sensitivity by controlling cell surface insulin receptor levels
Source: Nat Commun. 2016 Aug 31;7:12639. doi: 10.1038/ncomms12639 (PMC5013666; doi:10.1038/ncomms12639)
Supplement: Supplementary Information — Supplementary Figures 1-12 and Supplementary Tables 1-4 [file ncomms12639-s1.pdf]

Supplementary Fig. 1

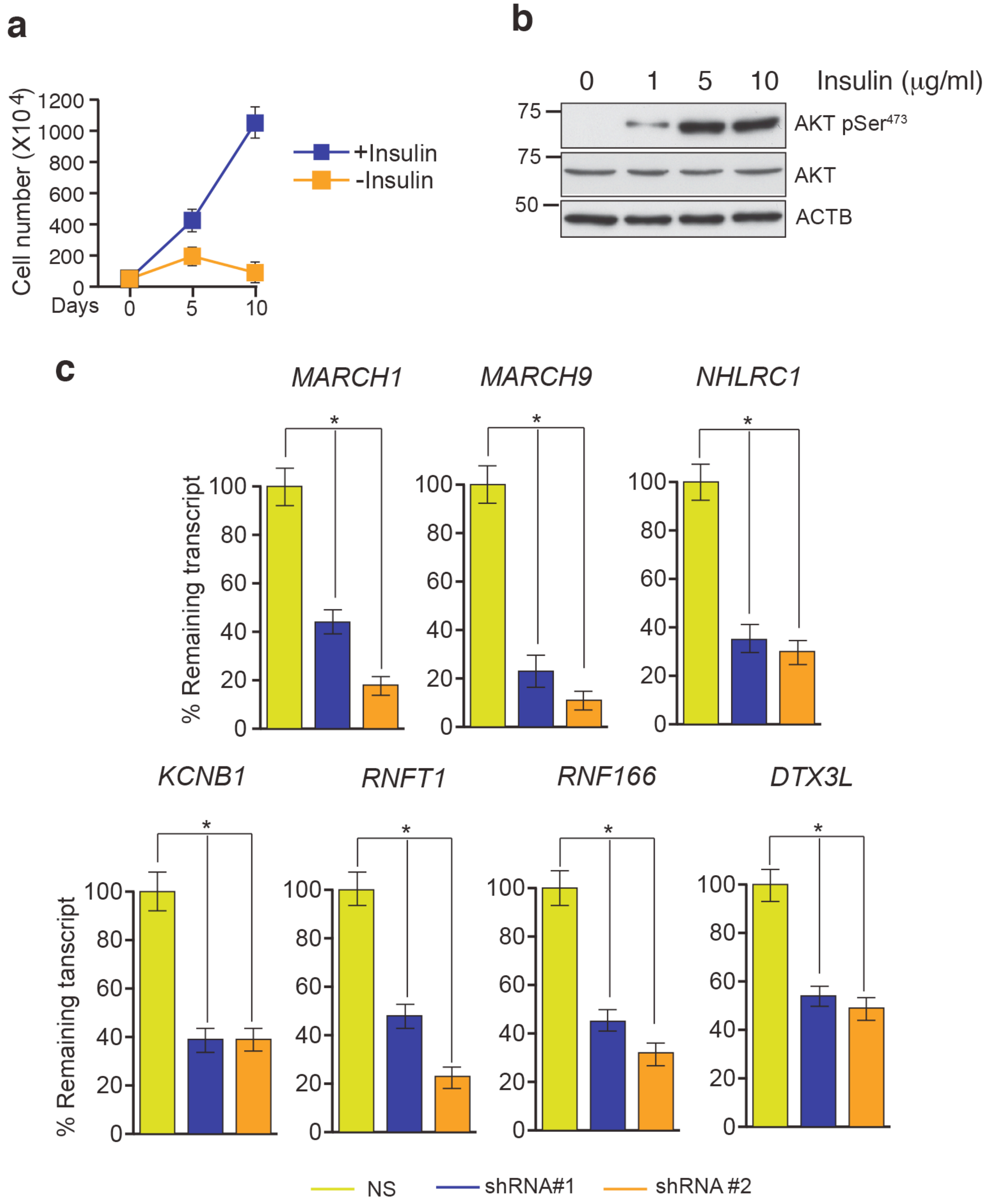

Supplementary Figure 1. HeLa cell survival, insulin signaling, and knockdown efficiency of identified candidates. (a) Cell proliferation, calculated using Trypan blue exclusion (n=3), for HeLa cells grown in serum free DMEM containing growth factors without insulin or 5 μg/ml insulin at days 5 and 10. **(b)** Insulin stimulation of AKT Ser^{473} phosphorylation. HeLa cells were serum starved overnight and then stimulated with indicated concentrations of insulin for 30 mins. **(c)** cDNA from HeLa cells expressing indicated shRNAs was analyzed by qRT-PCR (n=3). Knockdown efficiency relative to non-specific (NS) shRNA is shown. * compares NS shRNAs against indicated shRNAs. Data are mean ± SEM. In all panels, *P<0.05, comparisons by t-test.

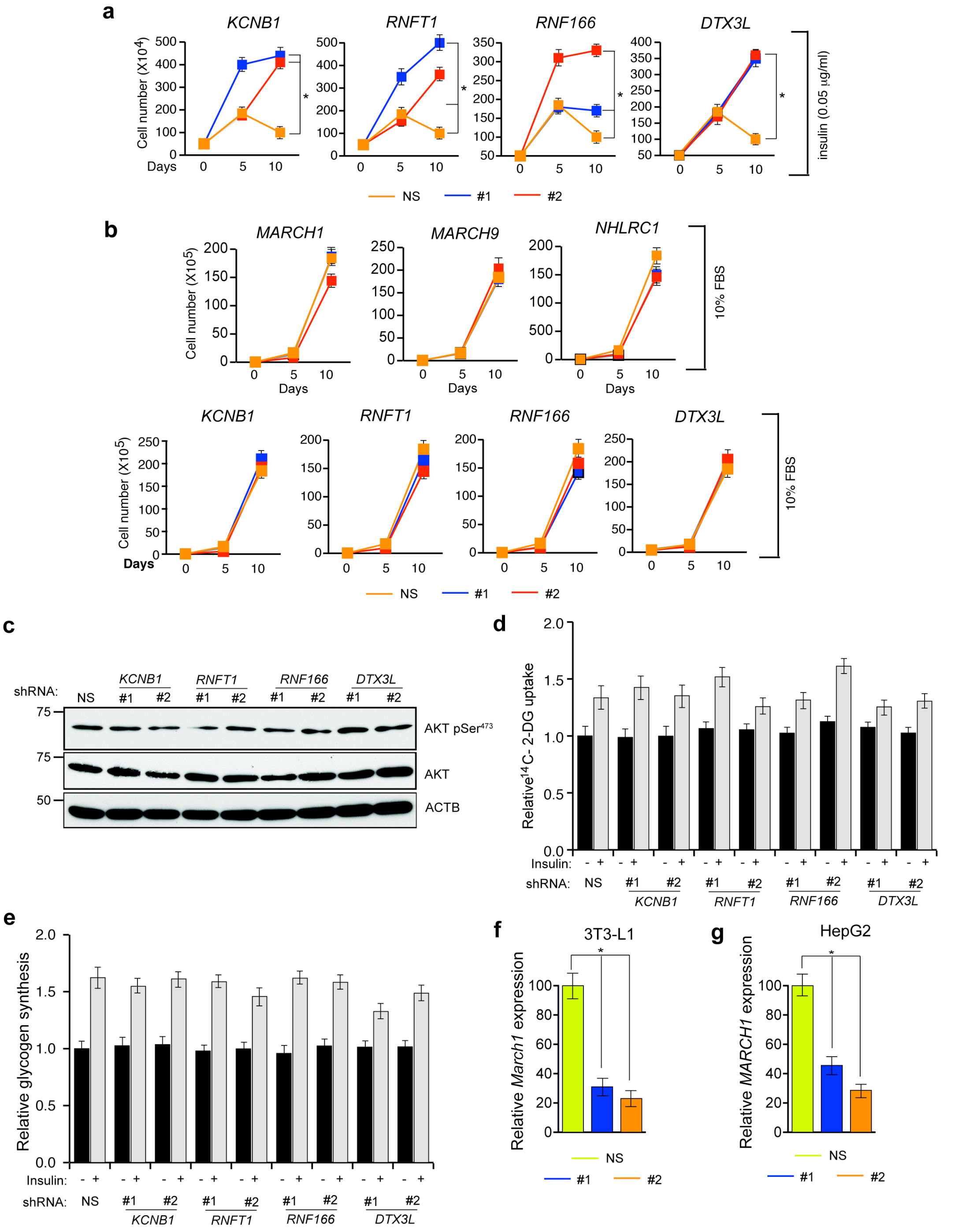

Supplementary Figure 2. Validation of candidates identified from the large-scale RNAi screen. (a) Relative cell number (n=3), calculated using Trypan blue exclusion, for HeLa cells expressing indicated shRNAs and grown in low-insulin medium at day 5 and 10. * compares NS and *MARCH1* shRNAs at day 10**.** **(b)** Relative cell number (n=3), calculated using Trypan blue exclusion, for HeLa cells expressing indicated shRNAs and grown in 10% serum-supplemented medium at days 5 and 10. **(c)** Immunoblot analysis of AKT Ser^{473} phosphorylation in HeLa cells expressing the indicated shRNAs. **(d)** Relative ^{14}C-2-deoxyglucose (2-DG) uptake (n=4) in HeLa cells expressing the indicated shRNAs. **(e)** Relative glycogen synthesis (n=3) in HeLa cells expressing the indicated shRNAs. **(f)** 3T3-L1 cells expressing *March1* or non-specific (NS) shRNA were analyzed for knockdown efficiency using qRT-PCR (n=3). **(g)** HepG2 cells expressing *MARCH1* or non-specific (NS) shRNA were analyzed for knockdown efficiency using qRT-PCR (n=3). Data are mean ± SEM. In all panels, *P<0.05, comparisons by *t*-test.

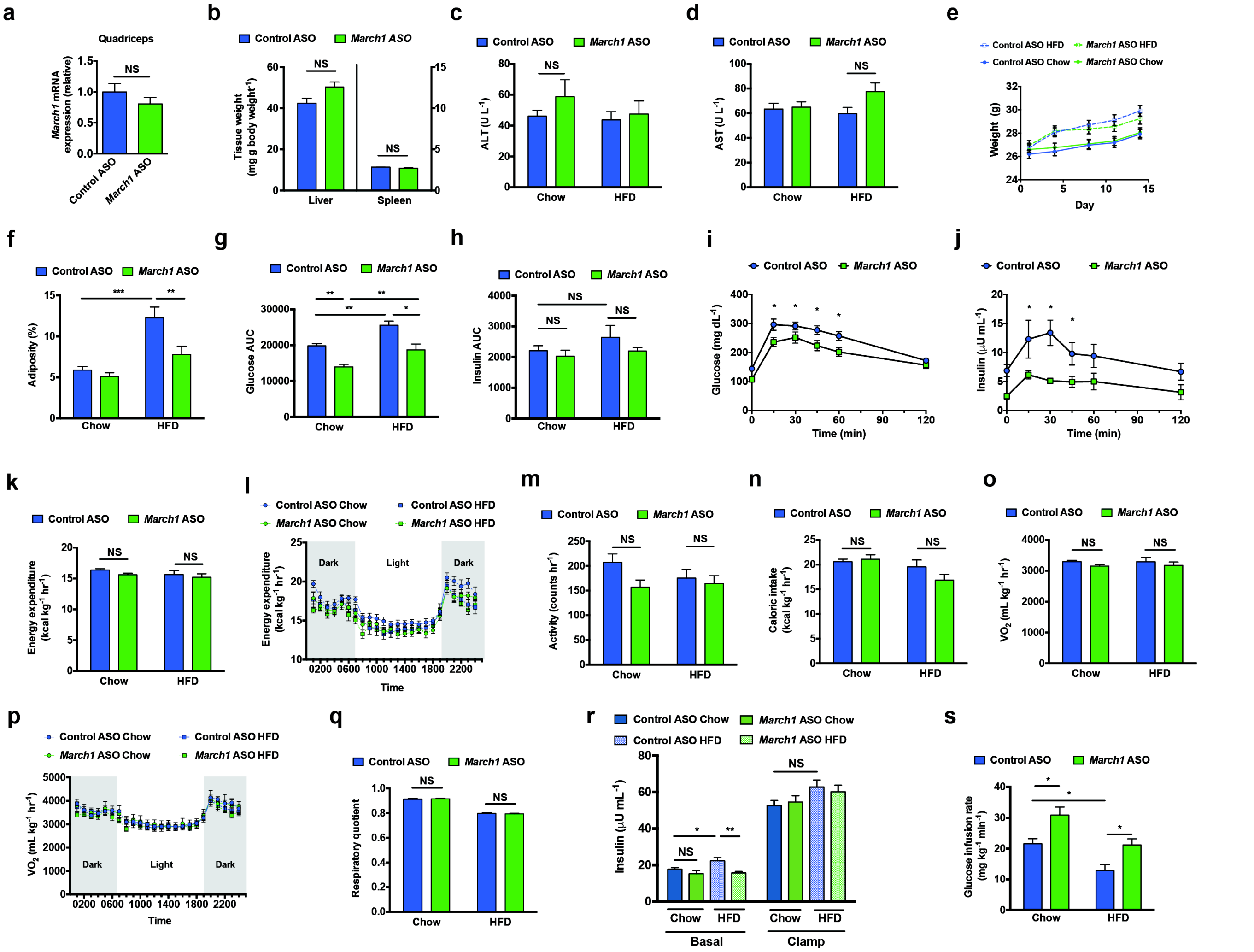

Supplementary Figure 3. Studies of male mice treated with *March1* ASO. (a-h) Mice were treated for two weeks with ASO and fed regular chow or HFD as indicated. **(a)** *March1* mRNA expression in quadriceps muscle. **(b)** Liver and spleen wet weight. **(c)** Plasma alanine transaminase (ALT) activity. **(d)** Plasma aspartate transaminase (AST) activity. **(e)** Body weight during ASO treatment. **(f)** Adiposity after ASO treatment measured by ^{1}H NMR. **(g)** Plasma glucose area under the curve during intraperitoneal glucose tolerance tests (ipGTTs) for data plotted in **Fig. 3b. (h)** Plasma insulin area under the curve during ipGTTs for data plotted in **Fig. 3c. (i-j)** Mice were treated with ASO for four weeks and fed regular chow. **(i)** Plasma glucose excursions during ipGTTs. **(j)** Plasma insulin excursions during ipGTTs. **(k-r)** Mice were treated with ASO for two weeks and fed chow or HFD as indicated before metabolic cage studies. **(k-l)** Whole-body energy expenditure. **(m)** Locomotor activity. **(n)** Caloric intake (**o-p)** Whole-body oxygen consumption. **(q)** Respiratory quotient. **(r-s)** Mice were treated with ASO for two weeks and fed regular chow or HFD as indicated before hyperinsulinemic-euglycemic clamp studies (see also **Fig. 3d-h**). **(r)** Plasma insulin levels before and during the clamp. **(s)** Mean glucose infusion rates during the steady-state period of the clamp for data plotted in **Fig. 3d**. Data are mean ± SEM. In all panels, *P<0.05, **P<0.005, ***P < 0.0005. In **(a-c)**, n = 6-7 mice per group; comparisons by two-way ANOVA. In **(e)**, n = 12-19 mice per group; comparisons by two-way ANOVA. In **(f)**, n = 18-23 mice per group; comparisons by two-way ANOVA. In **(g-h)**, n = 5-8 mice per group; comparisons by two-way ANOVA. In **(i-j)**, n = 8-9 mice per group; comparisons by two-tailed unpaired t-test. In **(k-q)**, n = 15-16 mice per group (regular chow) and 6-7 mice per group (HFD); comparisons by two-way ANOVA. In **(r-s)**, n = 9-11 mice per group; comparisons by two-way ANOVA.

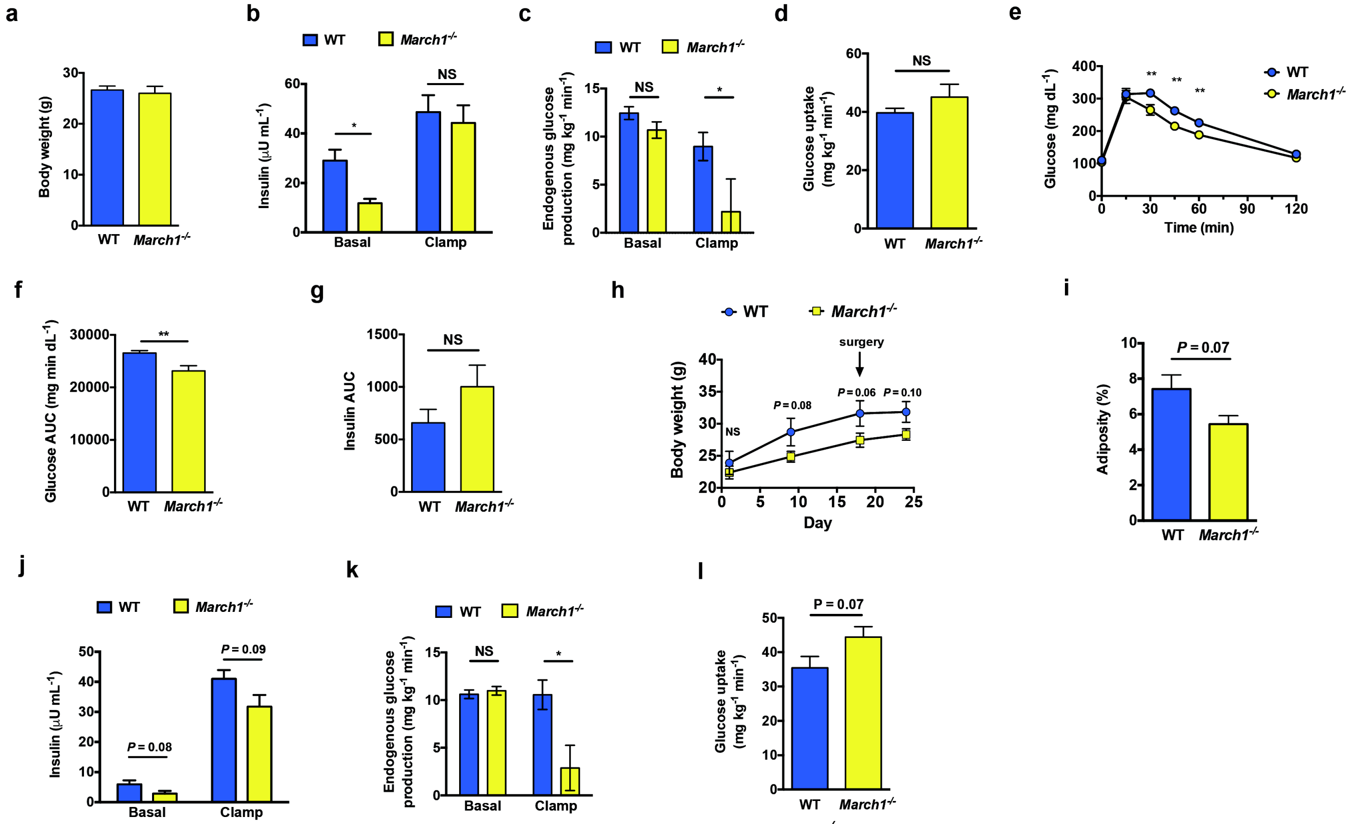

Supplementary Figure 4. Metabolic phenotyping of *March1*$^{-/-}$ mice. Littermate wild-type control mice were used in all studies of *March1*$^{-/-}$ mice. Different insulin infusion rates were used for studies of chow-fed and HFD-fed mice (2 mU kg^{-1} min^{-1} and 3 mU kg^{-1} min^{-1}, respectively), so data are not superimposed. **(a-d)** Hyperinsulinemic-euglycemic clamp studies were performed in chow-fed male mice (see also **Fig. 4a-c**). **(a)** Body weight. **(b)** Fasting and clamped plasma insulin concentrations. **(c)** Basal and clamped endogenous glucose production (EGP). **(d)** Whole-body glucose uptake during the steady-state period of the clamp. **(e-g)** Intraperitoneal glucose tolerance tests were performed in chow-fed female mice. **(e)** Plasma glucose excursions. **(f)** Plasma glucose area under the curve (AUC). **(g)** Plasma insulin AUC. **(h-l)** Hyperinsulinemic-euglycemic clamp studies were performed in HFD-fed mice (see also **Fig. 4d-f**). **(h)** Body weight during high-fat feeding. **(i)** Whole-body adiposity measured by ^{1}H NMR. **(j)** Fasting and clamped plasma insulin concentrations. **(k)** Basal and clamped EGP. **(l)** Whole-body glucose uptake during the steady-state period of the clamp. Data are mean ± SEM. In all panels, *P<0.05, **P<0.005; comparisons by two-tailed unpaired t-test. In **(a-d)**, n = 10 WT and 5 KO mice. In **(e-g)**, n = 11 mice per group. In **(h-l)**, n = 5 mice per group.

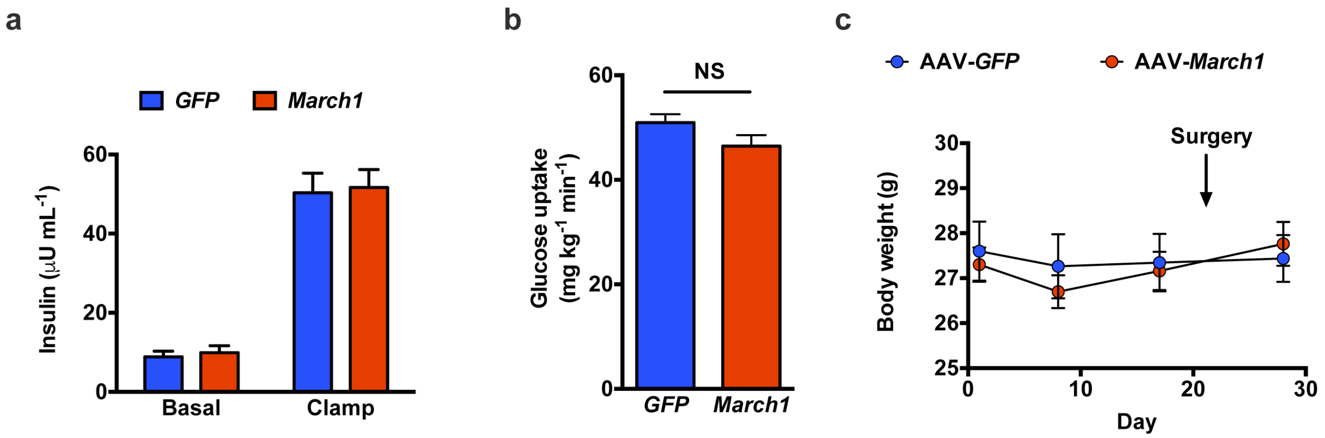

Supplementary Figure 5. Hyperinsulinemic-euglycemic clamp studies in mice treated with AAV-*March1*. Male 12-week old C57BL/6J mice were injected intravenously with AAV four weeks before study and fed regular chow. Hyperinsulinemic-euglycemic clamps were performed (see also **Fig. 5**). **(a)** Plasma insulin levels before and during the clamp. **(b)** Whole-body glucose uptake during the steady-state period of the clamp. **(c)** Body weight during treatment. Data are mean ± SEM. In all panels, *$P<0.05$. n = 7-9 mice per group; comparisons by *t*-test.

Supplementary Fig. 6

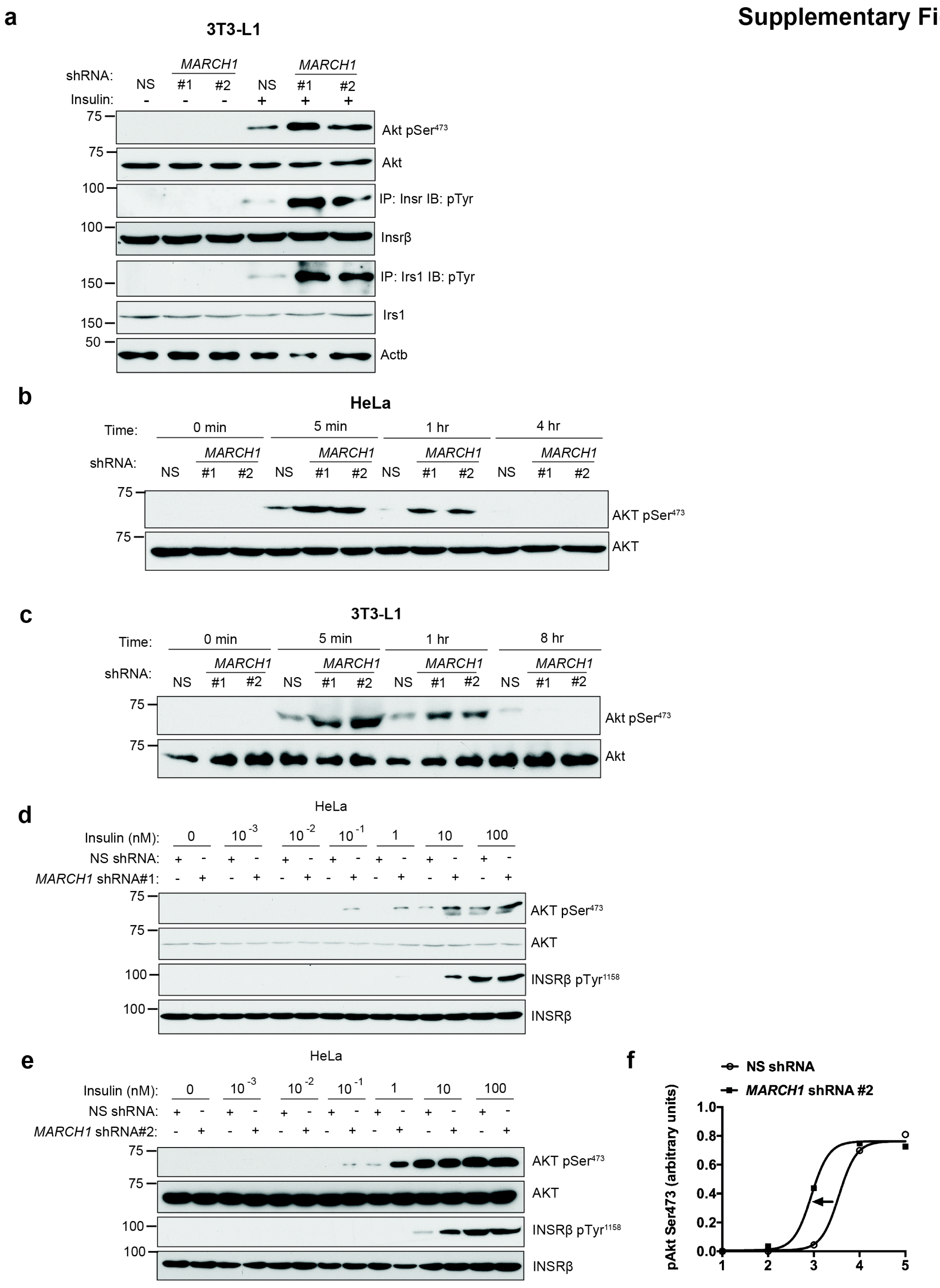

Supplementary Figure 6. Increased INSR signaling in cells with *MARCH1* knockdown. (a) Immunoblot analysis of insulin-stimulated INSR, IRS-1, and AKT activation in 3T3-L1 cells expressing the indicated shRNAs. **(b-c)** Insulin stimulation time course studies. HeLa cells **(b)** or 3T3-L1 cells **(c)** expressing the indicated shRNAs were serum starved overnight and stimulated with insulin (0.05 µg/mL) for the indicated duration. AKT Ser^{473} phosphorylation was assessed by immunoblotting. **(d-e)** Insulin dose-response studies. HeLa cells expressing the indicated shRNAs were serum starved overnight and stimulated with insulin at the indicated concentration for 30 min. AKT Ser^{473} and INSR Tyr^{1158} phosphorylation were assessed by immunoblotting. **(f).** Quantitation of the immunoblots in (**e**) fitted to insulin dose-response curves. See **Fig. 7c** for quantitation of the immunoblots in (**d**).

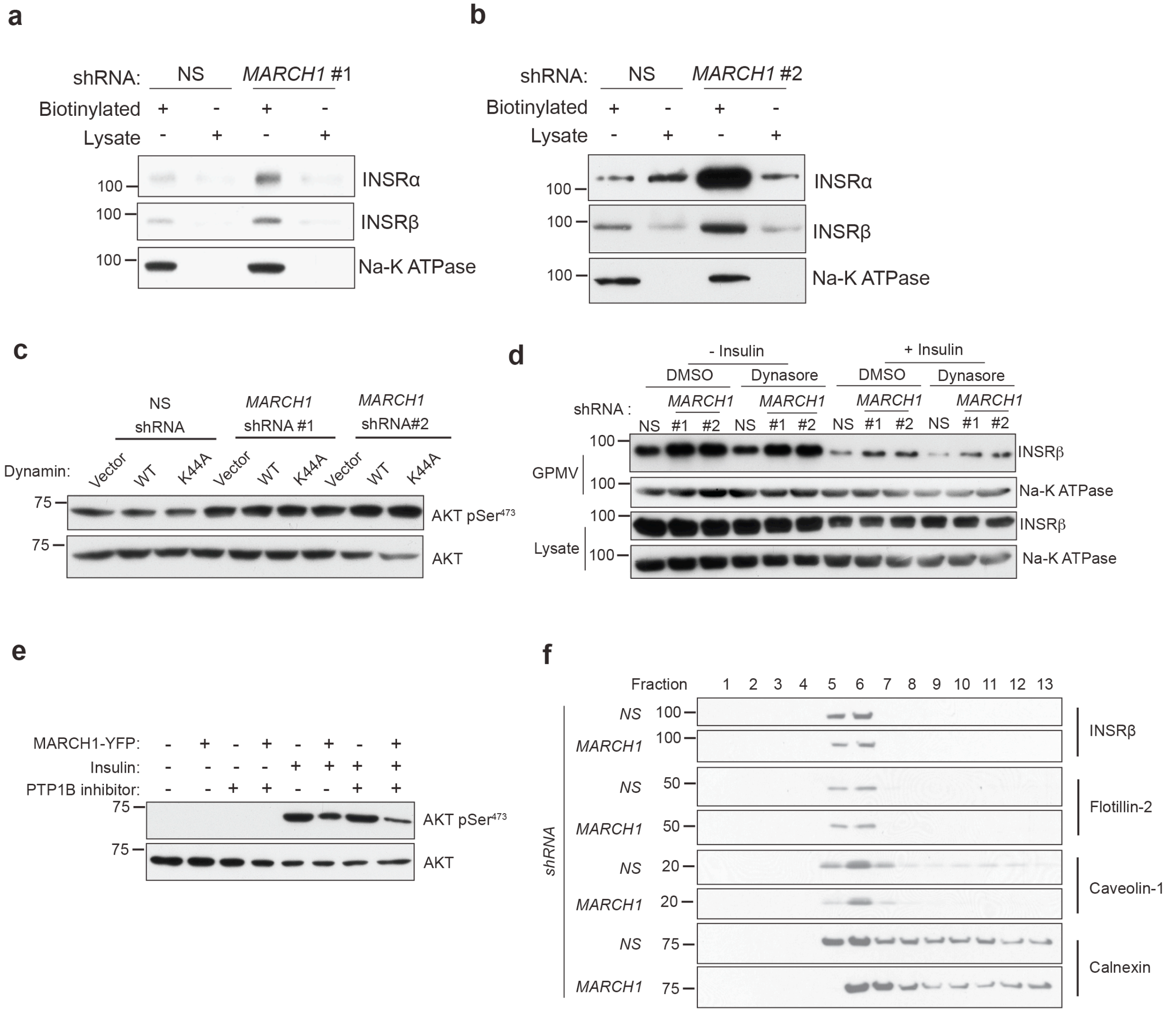

Supplementary Figure 7. MARCH1 regulates cell surface INSR expression without altering endocytosis, PTP1B activity, or membrane microdomain INSR content. (a-b) HeLa cells expressing the indicated shRNAs were serum starved and surface proteins were biotinylated. Avidin-isolated cell surface proteins and whole cell lysates were analyzed for the indicated proteins. See **Fig. 7d** for quantitation of multiple biotinylation experiments using *MARCH1* shRNA #1. **(c)** HeLa cells expressing indicated shRNAs were transfected with either empty vector (Vector), wild-type dynamin (WT) or endocytosis-defective K44A dynamin (K44A) and analyzed for AKT Ser^{473} phosphorylation. **(d)** HeLa cells expressing the indicated shRNAs and treated with or without the dynamin inhibitor Dynasore (20 μM) were harvested before or after 3 hr stimulation with 5μg/ml insulin. Immunoblot analysis of INSRβ in GPMVs or whole cell lysate was performed. Na-K ATPase is shown as a loading control. **(e)** HeLa cells were serum starved overnight and then stimulated with 0.05 μg/ml insulin. Samples were collected after 30 minute of insulin stimulation, either in the presence of DMSO or PTP1B inhibitor ($C_{26}H_{19}Br_2N_3O_7S_3$) (10 μM)**. (f)** Lipid rafts and caveolae were isolated by sucrose gradient fractionation from HeLa cells expressing the indicated shRNAs and were analyzed for marker proteins and INSRβ by immunoblot analysis.

Supplementary Fig. 8

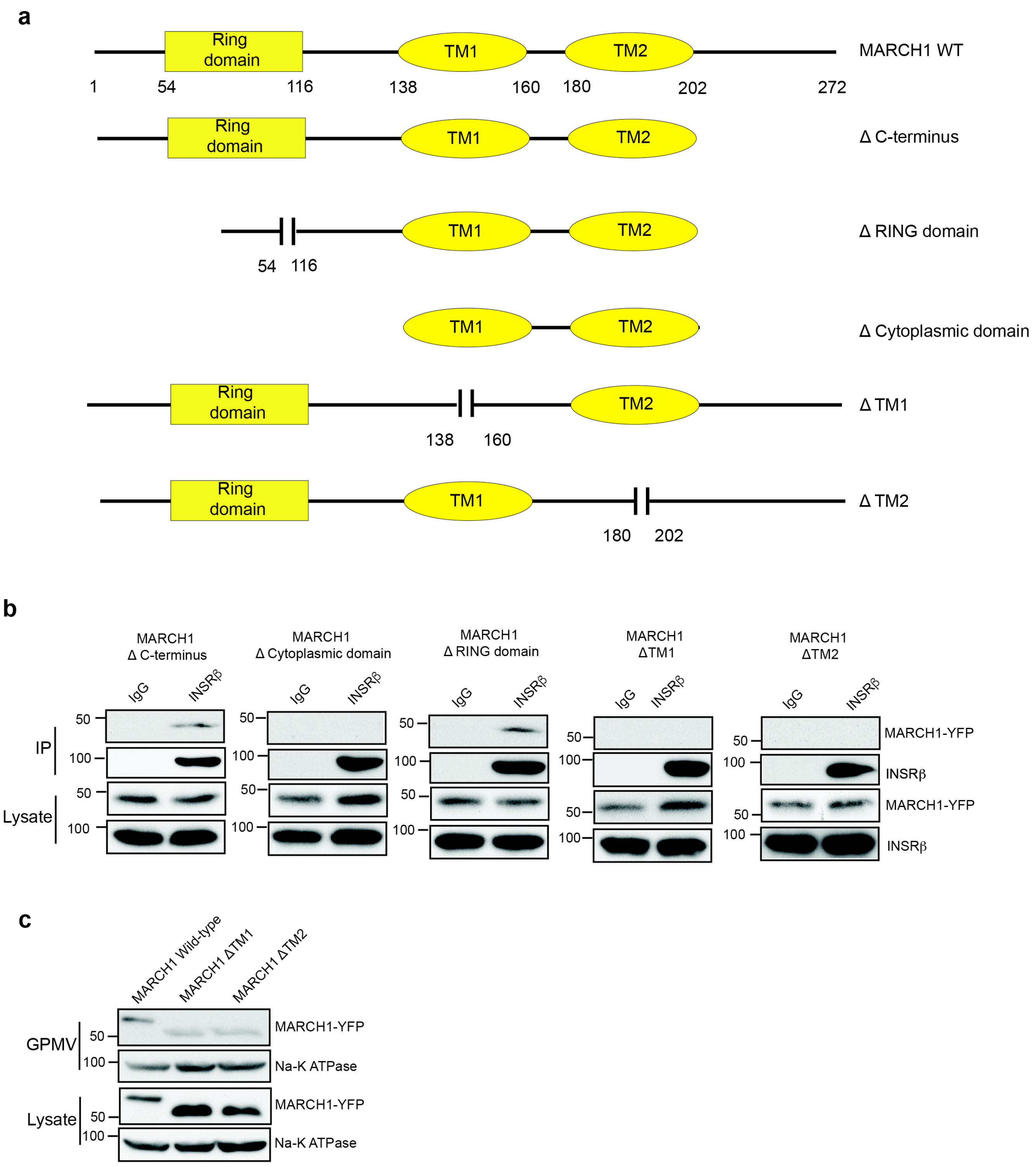

Supplementary Figure 8. (a) Schematic for the MARCH1 deletion mutants. **(b)** Indicated MARCH1 deletion mutants were transfected in HeLa cells along with INSRβ and immunoprecipitation was performed using either IgG or anti-INSRβ antibody. Both IP products and whole cell lysates were analyzed for indicated protein by immunoblotting. **(c)** Either wild type or indicated MARCH1 transmembrane deletion mutants were transfected in HeLa cells and GPMV and whole cell lysates were analyzed for indicated proteins by immunoblotting to assess MARCH1 localization.

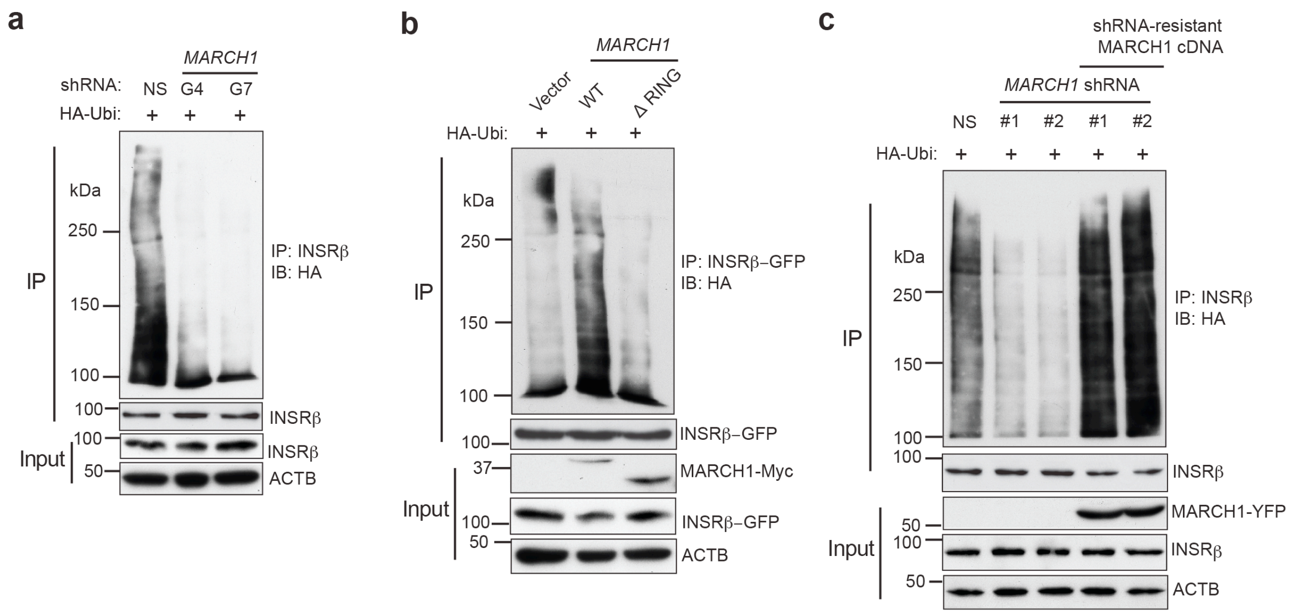

Supplementary Figure 9. MARCH1 ubiquitinates INSR. (a) Immunoblot analysis of INSRβ ubiquitination. INSRβ immunoprecipitated from HeLa cells expressing NS or *MARCH1* shRNAs and HA-ubiquitin was immunoblotted for HA. Lysates were probed for the indicated proteins. **(b)** Immunoblot analysis of INSRβ polyubiquitination. INSRβ immunoprecipitated from HeLa cells expressing *MARCH1-Myc* or *MARCH1-ΔRING-Myc* and HA-ubiquitin was immunoblotted for HA. Lysates were probed for indicated proteins. **(c)** Immunoblot analysis of INSRβ polyubiquitination. INSRβ immunoprecipitated from HeLa cells expressing NS or *MARCH1* shRNA #1 or #2 with or without shRNA-resistant *MARCH1* cDNA was immunoblotted for HA-ubiquitin. Lysates were probed for indicated proteins.

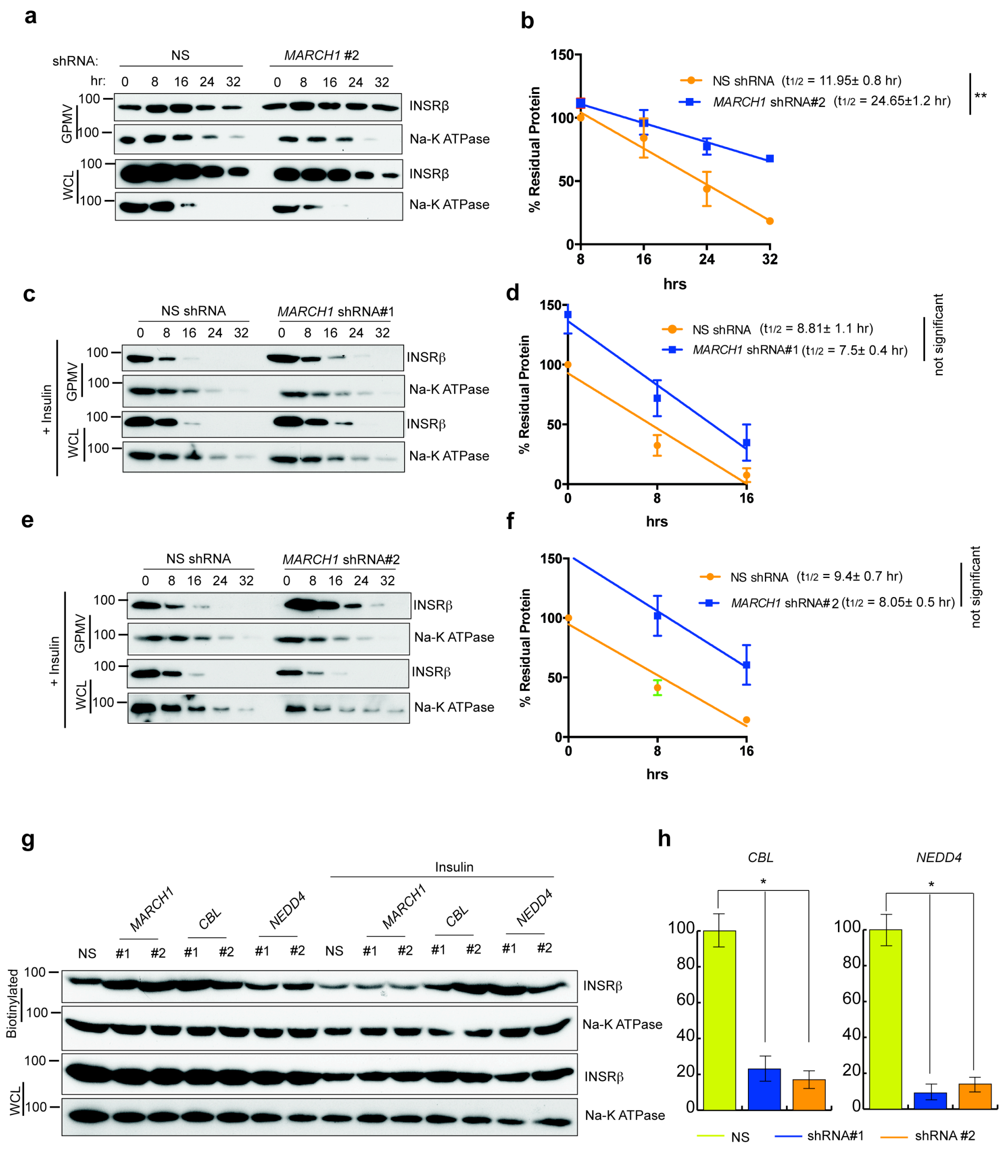

Supplementary Figure 10. MARCH1 regulates surface INSRβ half-life through direct ubiquitination. (a) HeLa cells expressing NS or *MARCH1* shRNA#2 were serum starved overnight and treated with cycloheximide (100 µg/ml). GPMVs and whole cell lysates (WCL) were collected at indicated time points and analyzed for indicated proteins by immunoblotting. **(b)** Quantitation of the immunoblots (n=3) shown in (a). **(c)** HeLa cells expressing indicated shRNAs were serum starved overnight and treated with insulin (5 µg/ml) and cycloheximide (100 µg/ml). GPMVs and whole cell lysates (WCL) were collected at indicated time points and analyzed for indicated proteins by immunoblotting. **(d)** Quantitation of the immunoblots (n=3) shown in (c). **(e)** HeLa cells expressing indicated shRNAs were serum starved overnight and treated with insulin (5 µg/ml) and cycloheximide (100 µg/ml). GPMVs and whole cell lysates (WCL) were collected at indicated time points and analyzed for indicated proteins by immunoblotting. **(f)** Quantitation of the immunoblots (n=3) shown in (e). **(g)** Biotinylation assay. HeLa cells expressing the indicated shRNA were serum starved overnight and left untreated or insulin treated (5 µg/ml) for 3 hrs. The biotinylated fraction and whole cell lysates were analyzed for INSRβ and Na-K ATPase content. **(h)** HeLa cells expressing non-specific (NS) or *CBL* or *NEDD4* shRNA were analyzed for knockdown efficiency using qRT-PCR (n=3). Data are mean ± SEM. *P<0.05 and **P<0.05, comparisons by t-test.

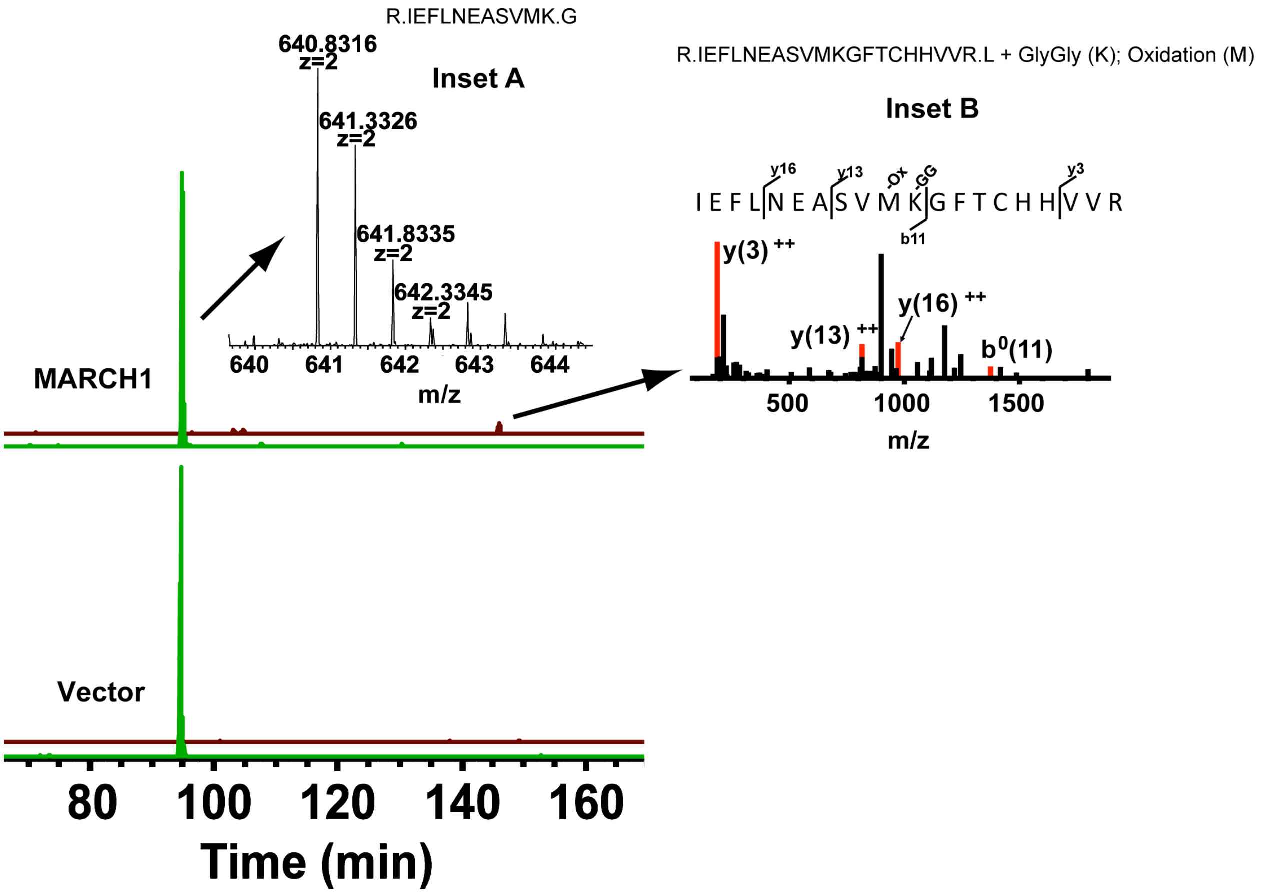

Supplementary Figure 11. Mass spectrometry analysis of INSRβ in HeLa cells transfected with vector or MARCH1. Extracted ion chromatograms are shown for the non-modified (green) and modified (red) INSRβ peptide derived from vector- (lower) and *MARCH1*- (upper) transfected HeLa cells. Inset A shows the averaged mass spectrum corresponding to the non-modified peptide. Inset B indicates the corresponding MS/MS fragmentation of the modified peptide at m/z 816.4086 with assigned b- and y- fragment ions. Above the MS/MS spectrum is the modified peptide sequence indicating the location of the identified modified lysine residue. Note that methionine is oxidized.

Supplementary Fig. 12

Figure 1c

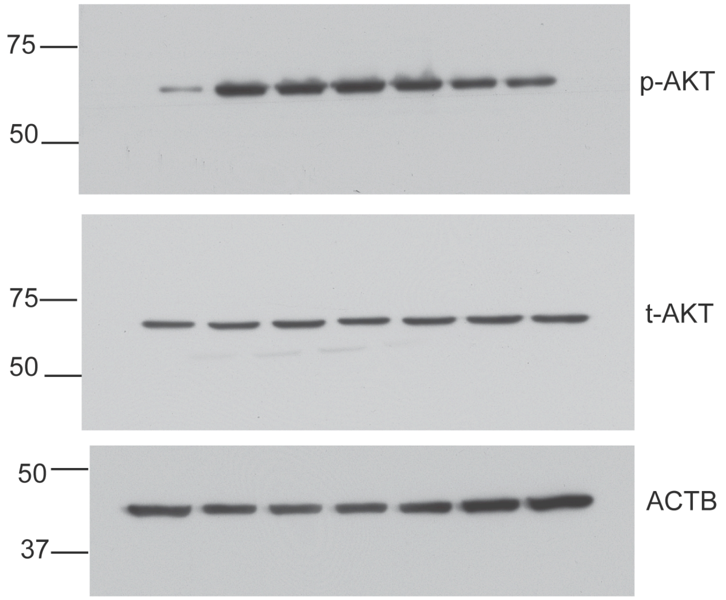

Figure 2c

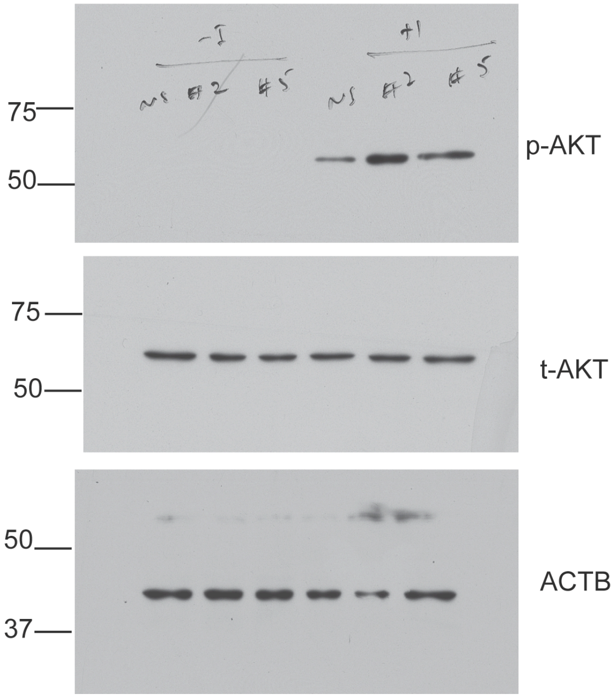

Figure 2f

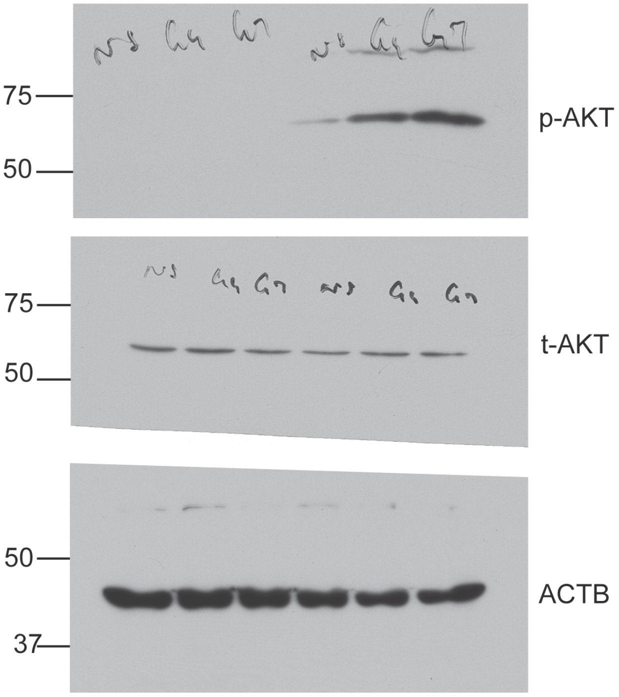

Figure 5a

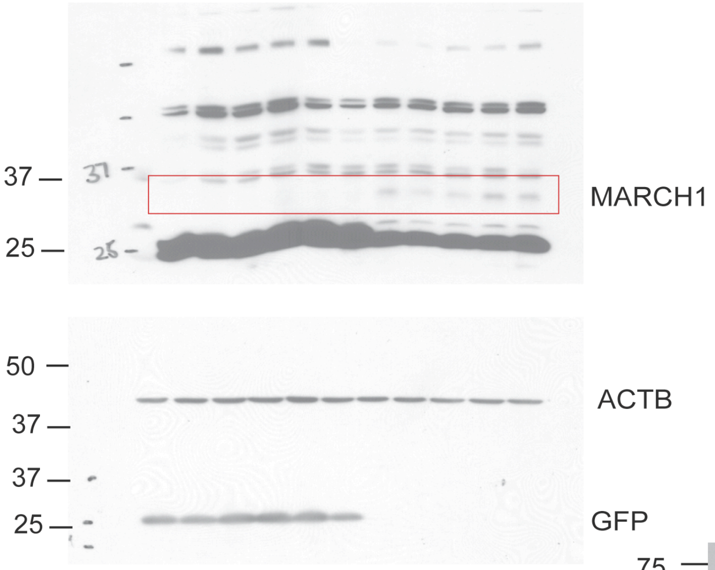

Figure 7a

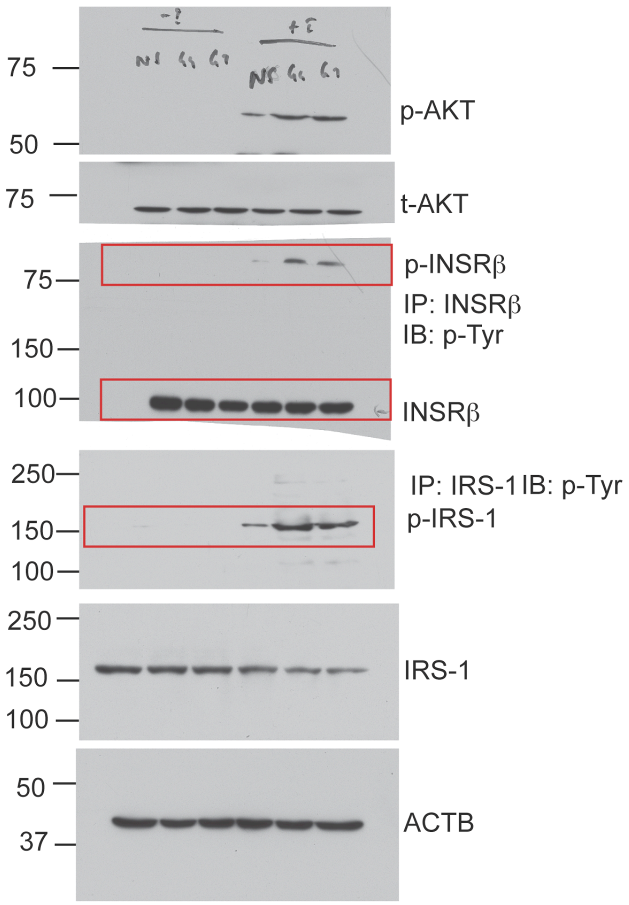

Figure 7b

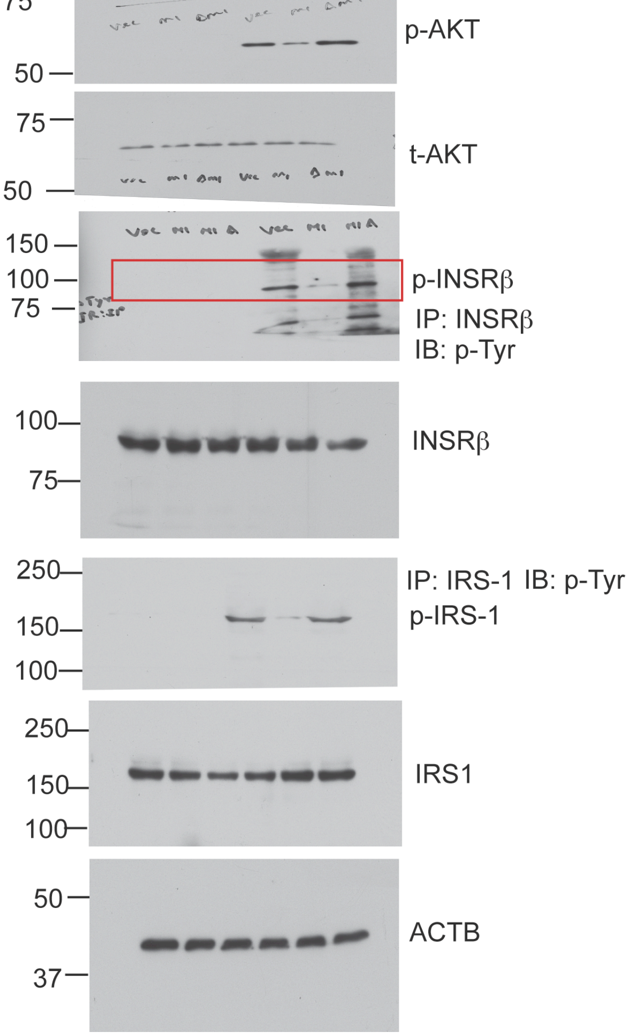

Figure 8a

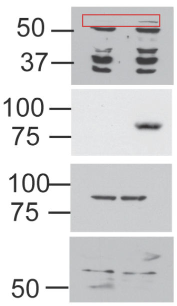

Figure 8a

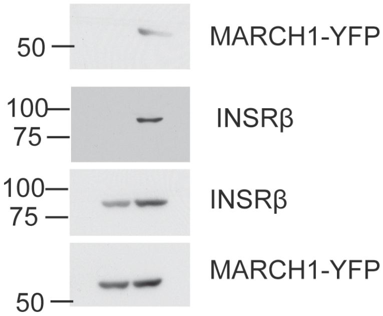

Figure 8b

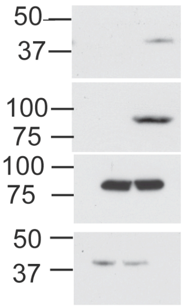

Figure 8b

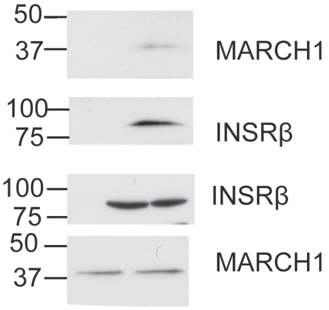

FIgure 8c

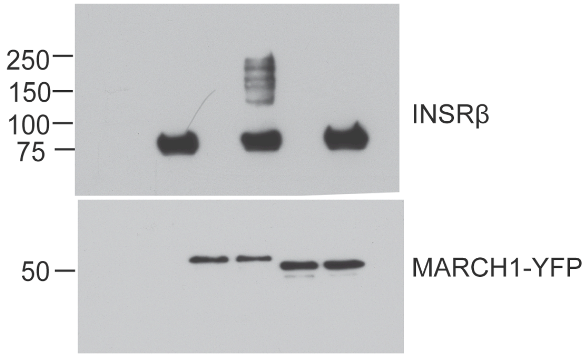

FIgure 8d

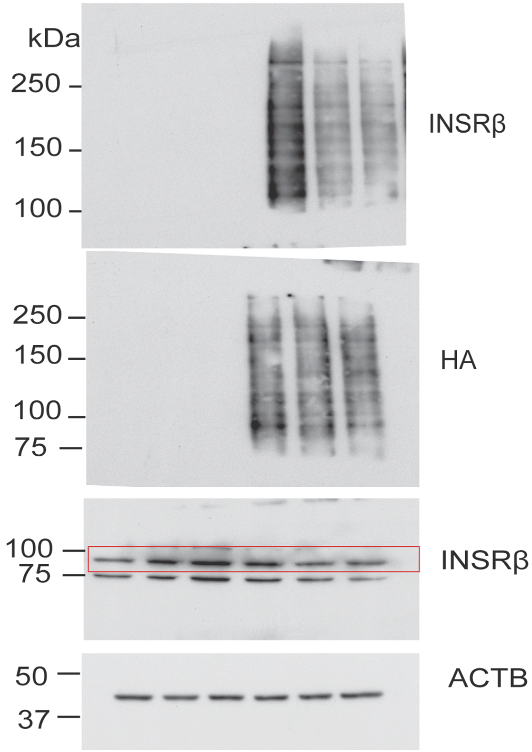

FIgure 8e

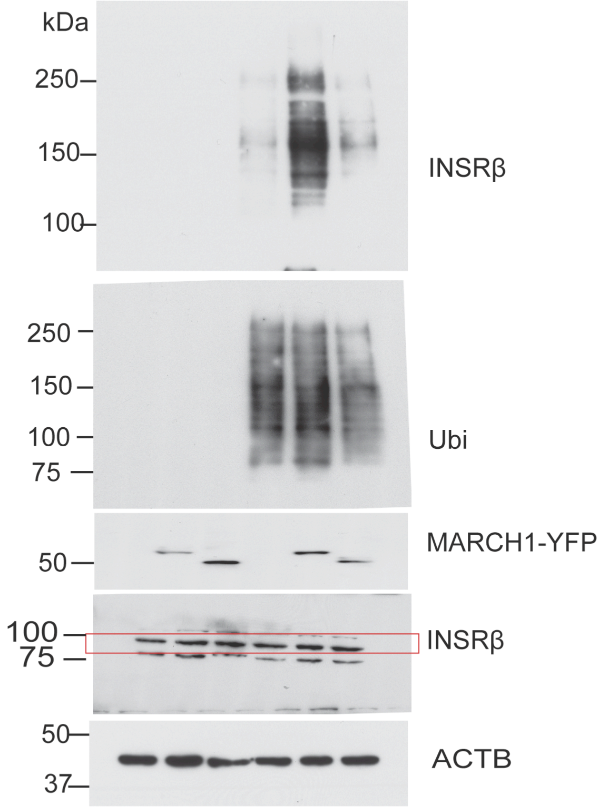

Figure 8f

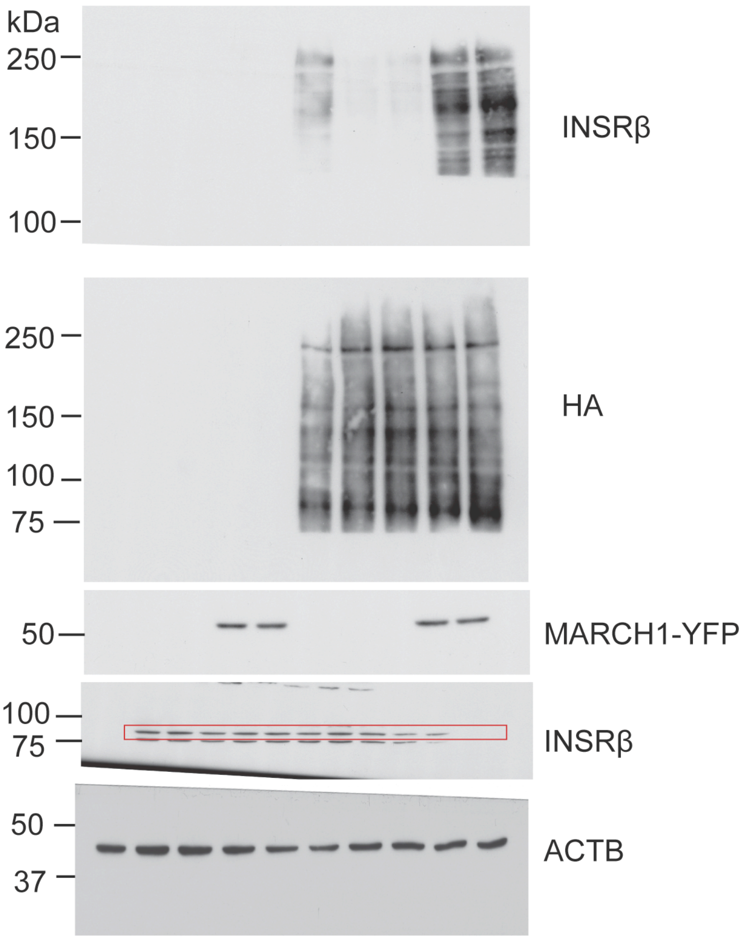

Figure 8g

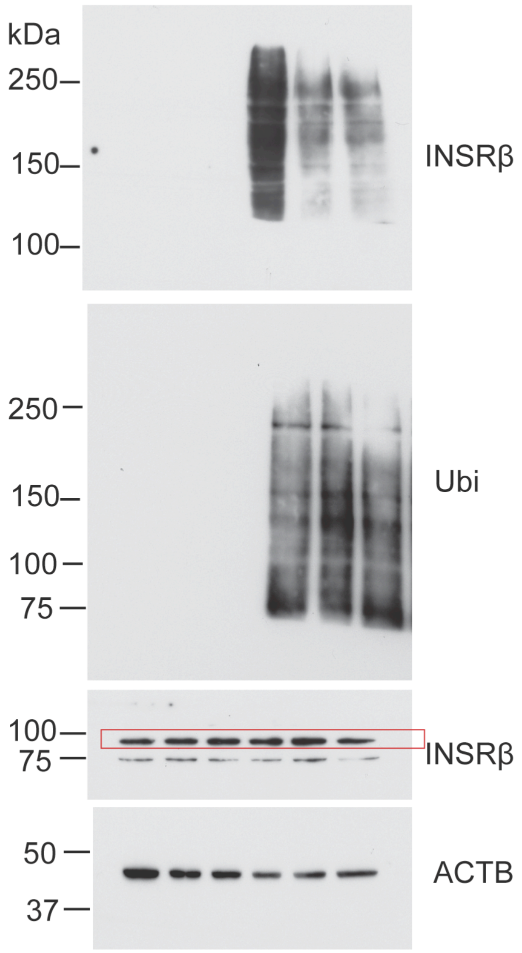

Figure 8h

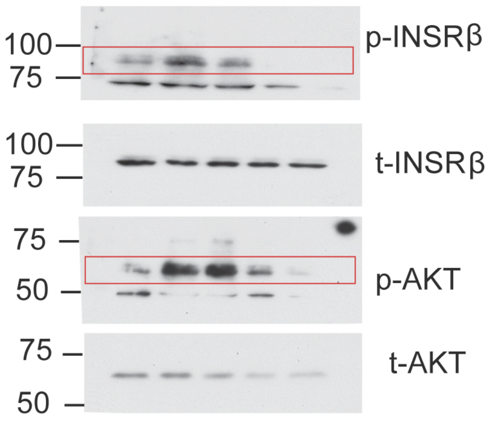

Figure 9a

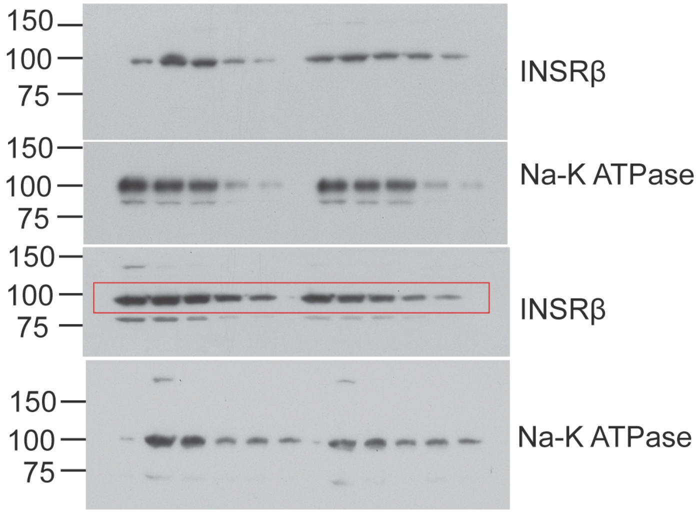

Figure 9c

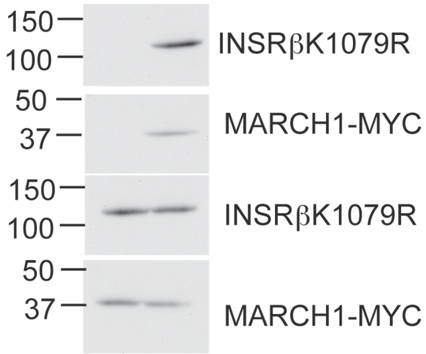

Figure 9d

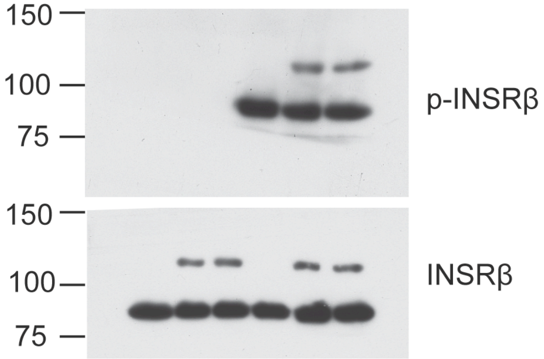

Figure 9e

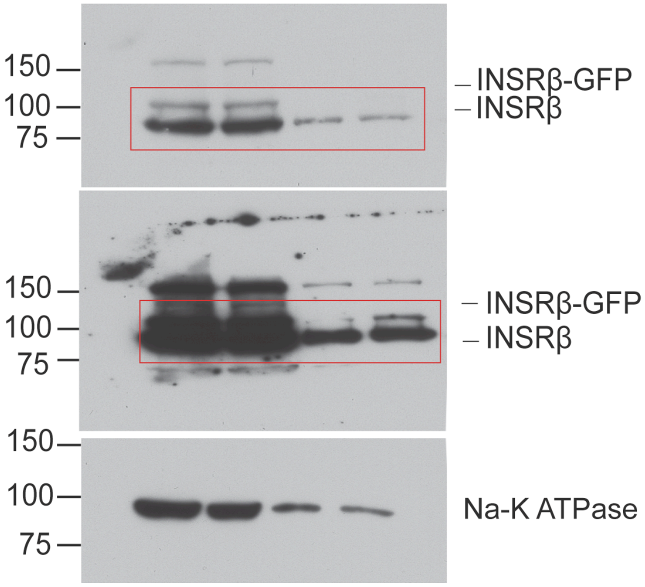

Figure 9g

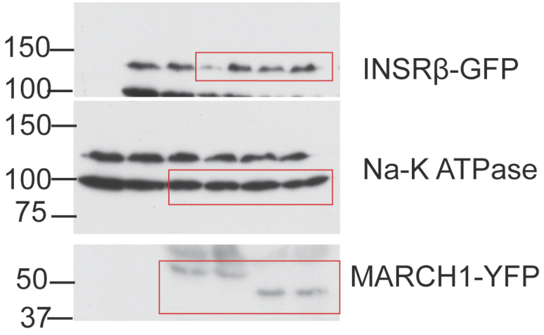

Suppl. Fig. 1b

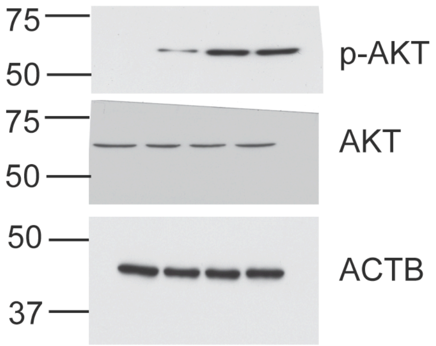

Suppl. Fig. 2c

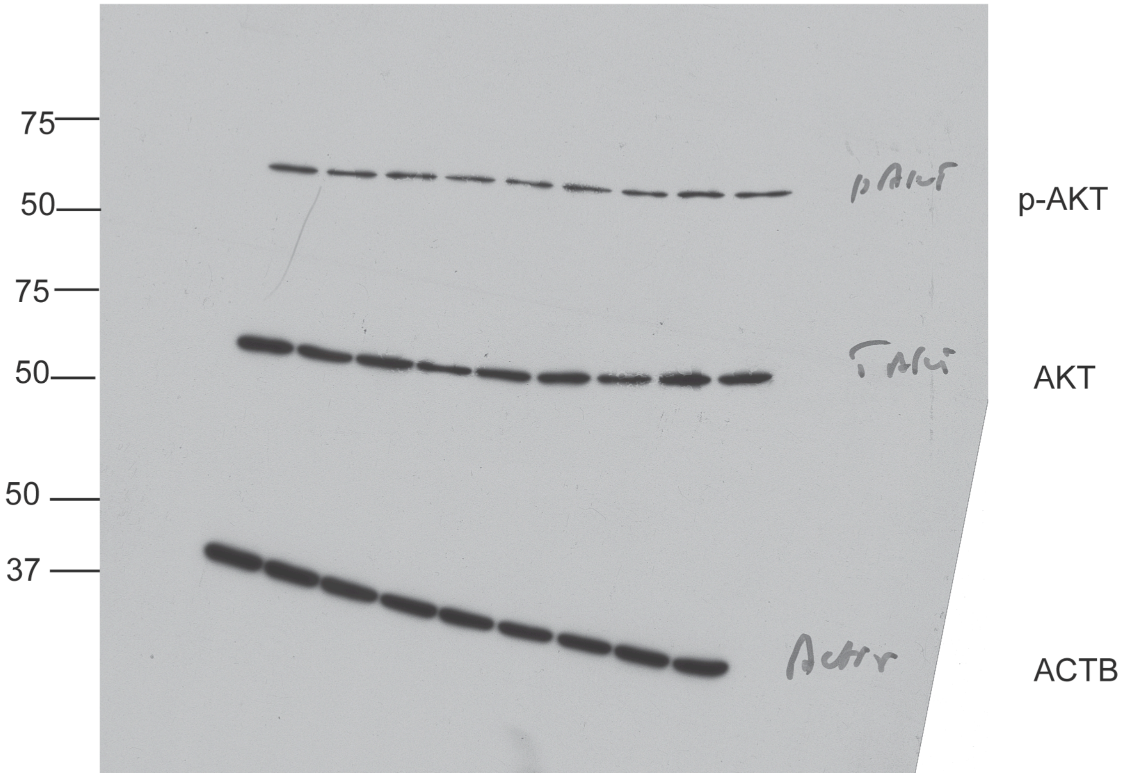

Suppl. Fig. 6a

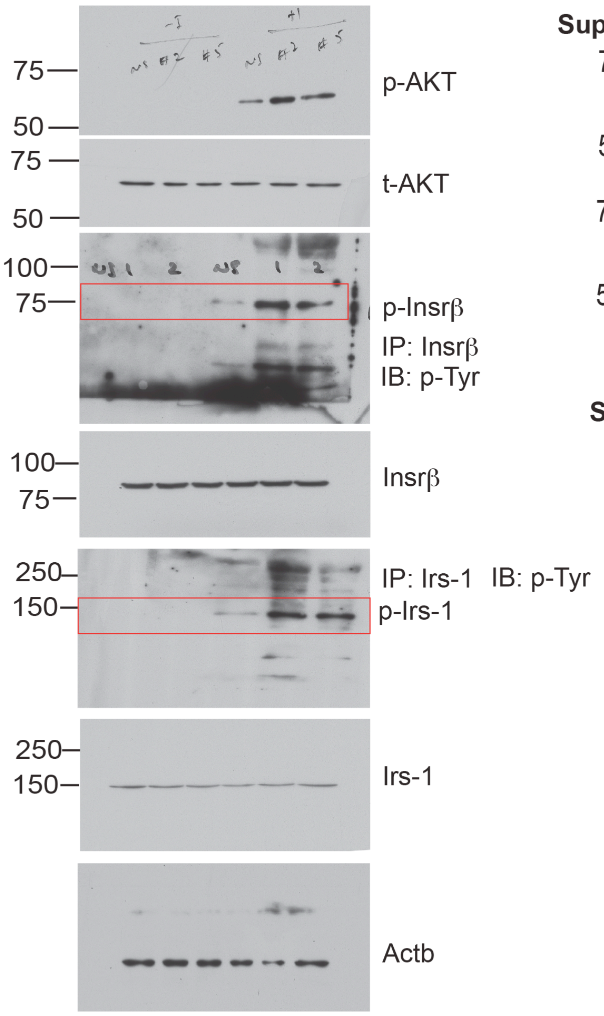

Suppl. Fig. 6b

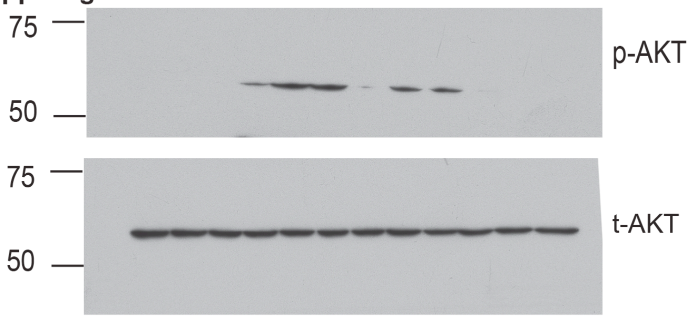

Suppl. Fig. 6c

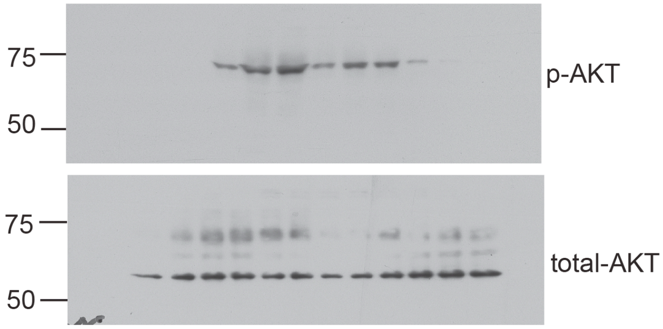

Suppl. Fig. 6e

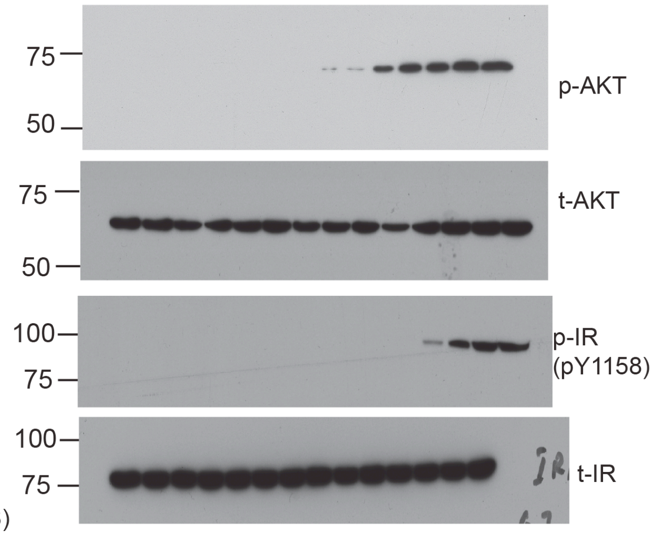

Suppl. Fig. 6d

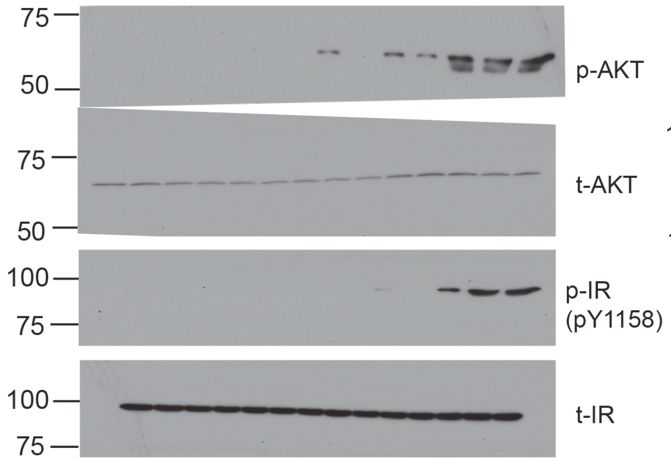

Suppl. Fig. 7a

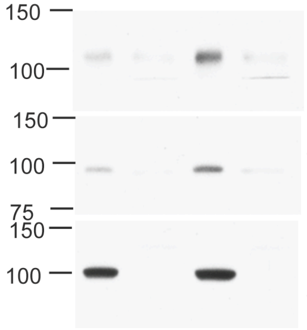

Suppl. Fig. 7b

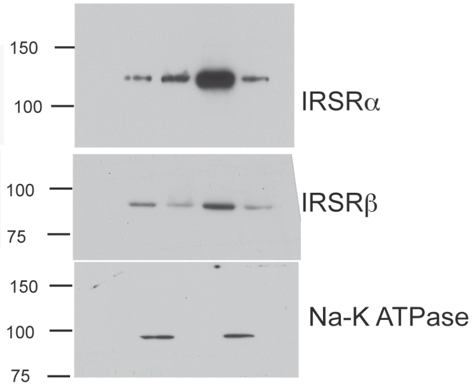

Suppl. Fig. 7c

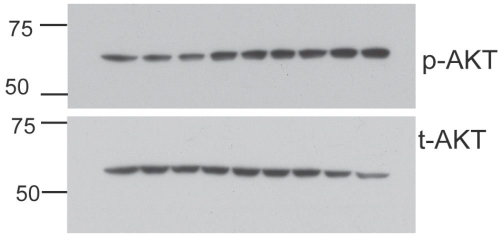

Suppl. Fig. 7f

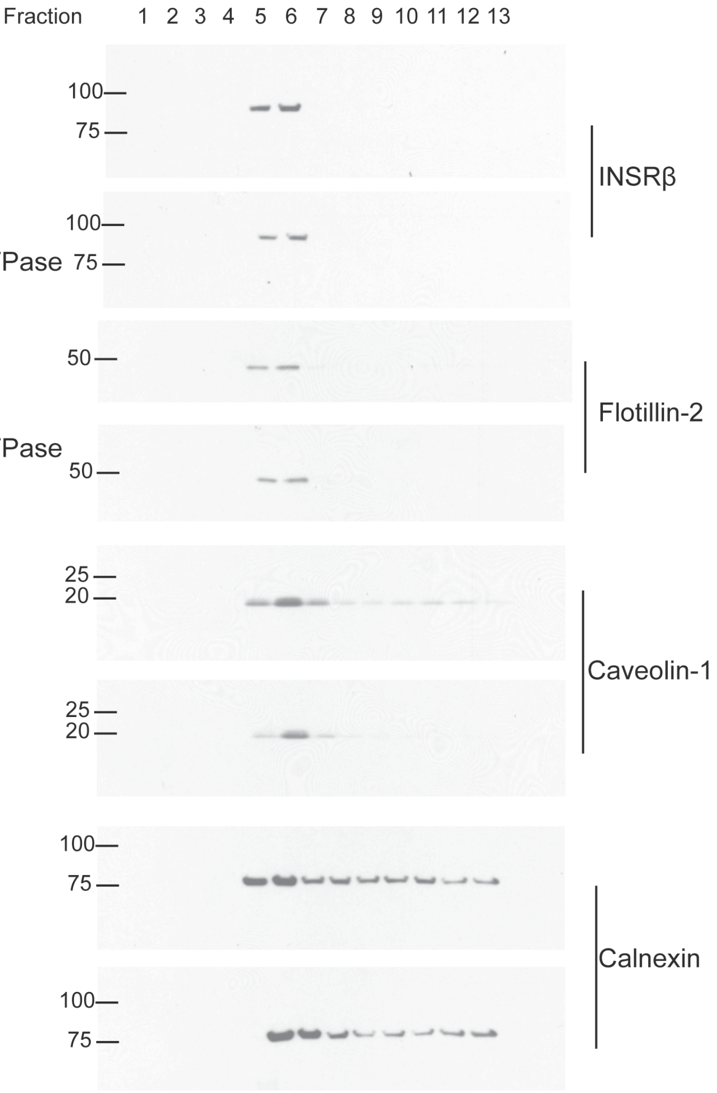

Suppl. Fig. 7d

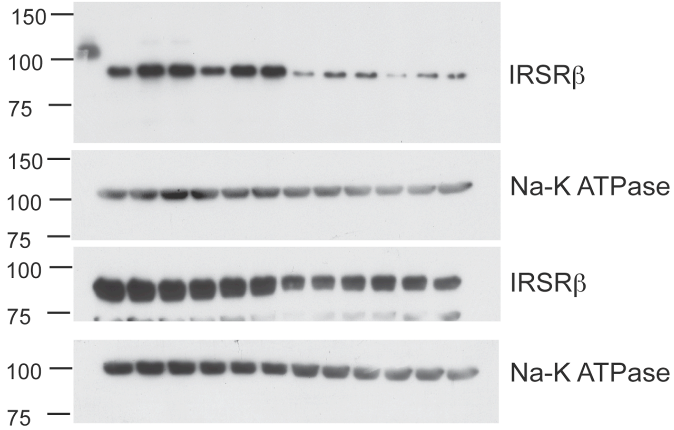

Suppl. Fig. 7e

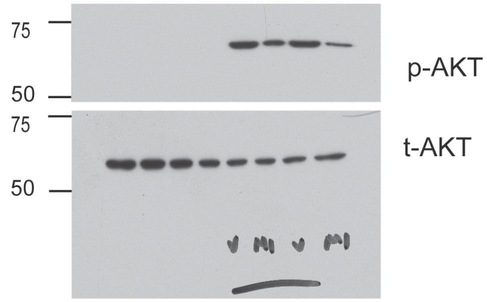

Suppl. Fig. 8b

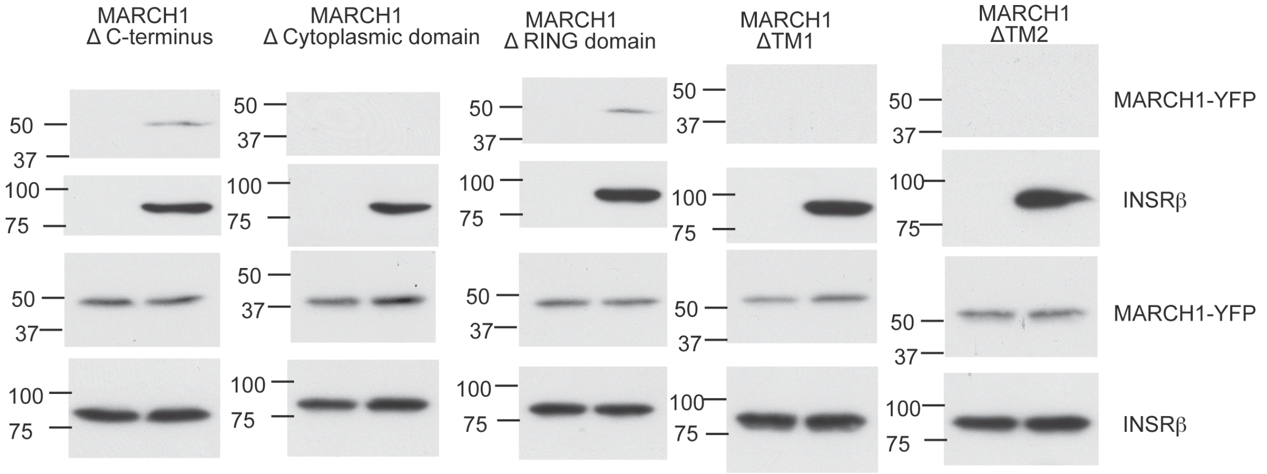

Suppl. Fig. 8c

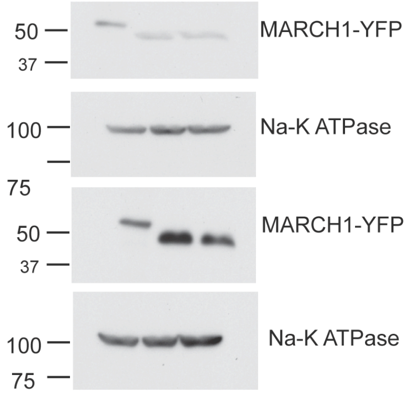

Suppl. Fig. 9a

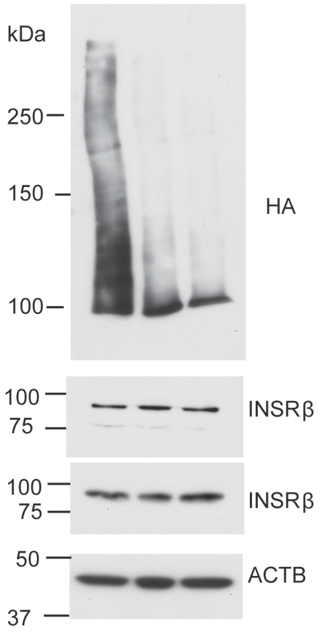

Suppl. Fig. 9b

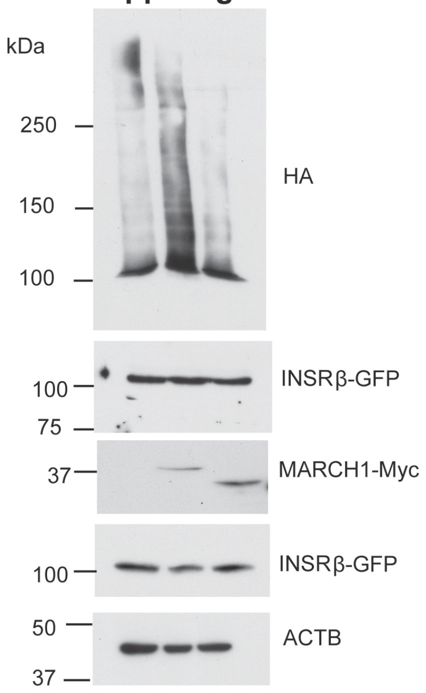

Suppl. Fig. 9c

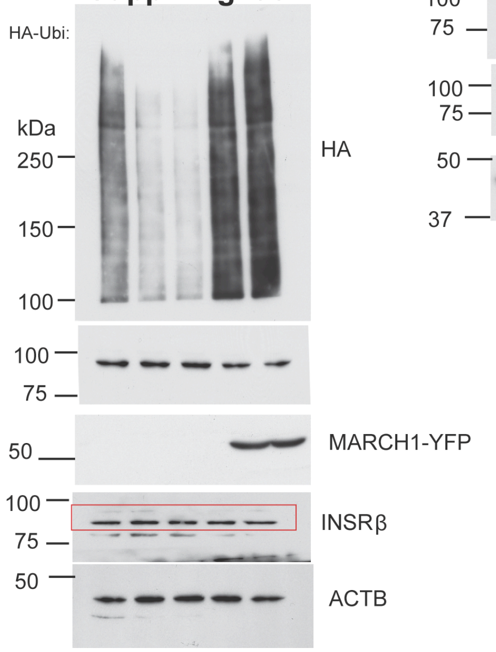

Suppl. Fig. 10a

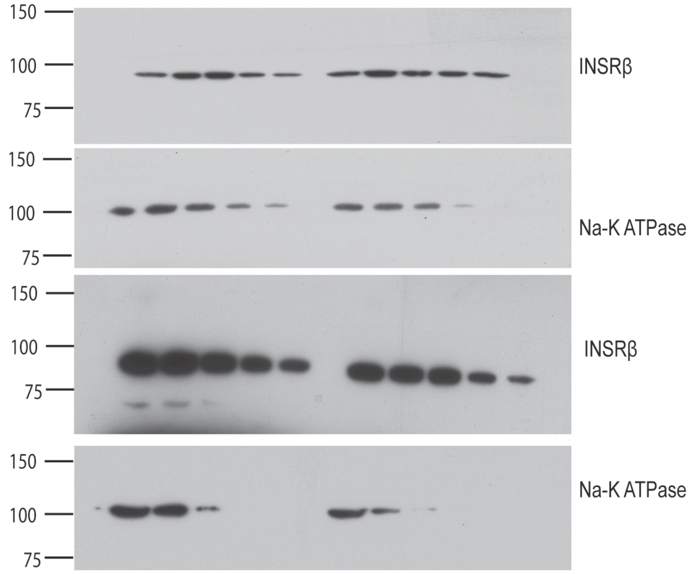

Suppl. Fig. 10c

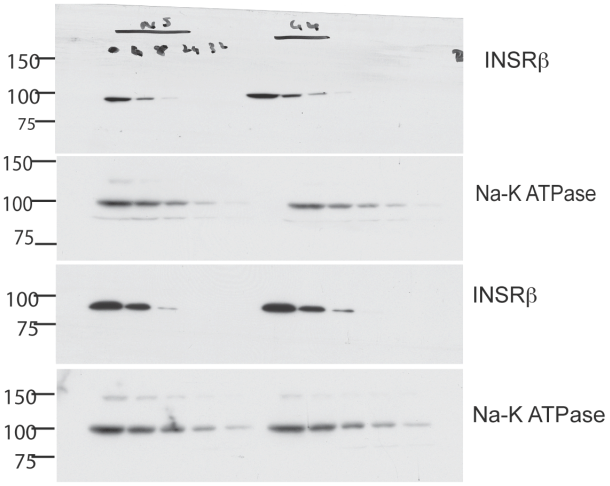

Suppl. Fig. 10e

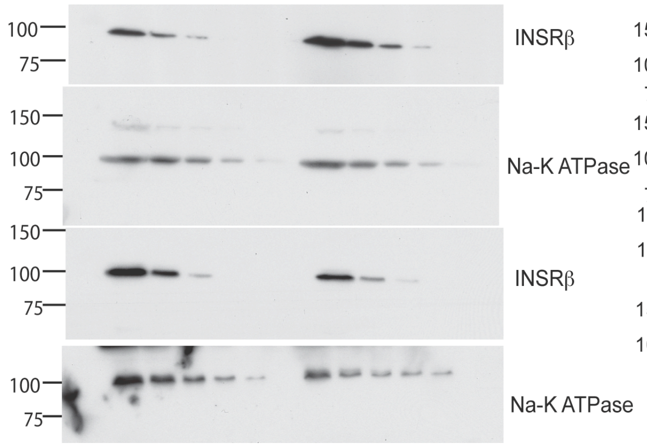

Suppl. Fig. 10g

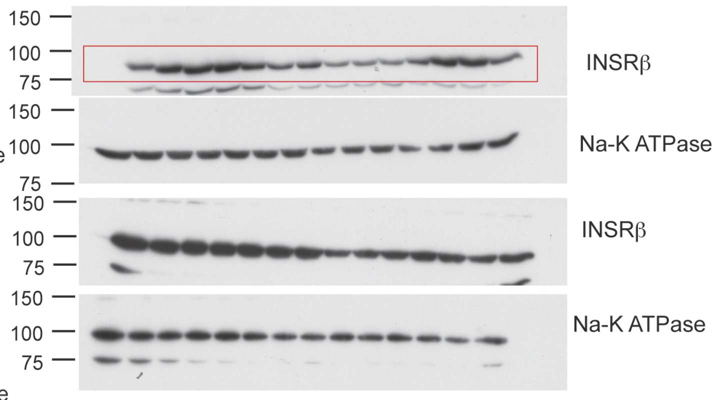

Supplementary Figure 12. Uncropped images of western blot analysis.

Supplementary Table 1. List of E3 ligase/adapter genes targeted by the shRNAs in the large-scale RNAi screen

Gene Name	Accession Number
ANKIB1	NM_019004
ANAPC11	NM_001002244 NM_001002245 NM_001002246 NM_001002247 NM_001002248 NM_001002249 NM_016476
ARIH1	NM_005744
ARIH2	NM_006321
AMFR	NM_001144
'MARCH7	NM_022826
BIRC2	NM_001166
BIRC3	NM_001165 NM_182962
BIRC4	NM_001167
BIRC7	NM_022161 NM_139317
BIRC8	NM_033341
BFAR	NM_016561
BMI1	NM_005180
BRAP	NM_006768
BARD1	NM_000465
BRCA1	NM_007294 NM_007295 NM_007296 NM_007297 NM_007298 NM_007299 NM_007300 NM_007302 NM_007303 NM_007304 NM_007305
CBL	NM_005188
CBLB	NM_170662
CBLC	NM_012116
CBLL1	NM_024814
CNOT4	NM_001008225 NM_013316
CGRRF1	NM_006568
CHFR	NM_018223
C1orf164	NM_018150
C13orf7	NM_024546
C14orf4	NM_024496
C16orf28	NM_023076
KIAA1542	NM_020901
CCNB1IP1	NM_021178 NM_182849 NM_182851 NM_182852
DPF1	NM_004647
DCST1	NM_152494
DTX3	NM_178502
DTX3L	NM_138287
DTX4	NM_015177
DTX1	NM_004416
DTX2	NM_020892
FANCL	NM_018062
GTF2H2	NM_001515
HLTF	NM_003071 NM_139048
IBRDC1	NM_152553
IBRDC2	NM_182757

IBRDC3	NM_153341
LRSAM1	NM_001005373 NM_001005374 NM_138361
LNX1	NM_032622
LNX2	NM_153371
LINCR	NM_001080535
LONRF1	NM_152271
LONRF2	NM_198461
LONRF3	NM_001031855 NM_024778
MGRN1	NM_015246
MKRN1	NM_013446
MKRN2	NM_014160
MKRN3	NM_005664
MSL2L1	NM_018133
MDM2	NM_002392 NM_006878 NM_006879 NM_006881 NM_006882
MDM4	NM_002393
'MARCH1	NM_017923
'MARCH2	NM_001005415 NM_001005416 NM_016496
'MARCH3	NM_178450
'MARCH4	NM_020814
'MARCH5	NM_017824
'MARCH6	NM_005885
'MARCH8	NM_001002265 NM_001002266 NM_145021
'MARCH9	NM_138396
MNAT1	NM_002431
MID1	NM_033291 NM_000381 NM_001098624 NM_033290
MID2	NM_012216 NM_052817
MIB1	NM_020774
MIB2	NM_080875
MAP3K1	XM_001128827 XM_042066
C1orf166	NM_024544
MYCBP2	NM_015057
MYLIP	NM_013262
NEURL	NM_004210
NHLRC1	NM_198586
NSMCE1	NM_145080
NFX1	NM_002504 NM_147133 NM_147134
NFXL1	NM_152995
OSTM1	NM_014028
PARC	NM_015089
PARK2	NM_004562 NM_013987 NM_013988
PDZRN3	NM_015009
PDZRN4	NM_013377
PEX12	NM_000286
PXMP3	NM_000318 NM_001079867
PEX10	NM_002617 NM_153818
PHF7	NM_016483 NM_173341

PCGF1	NM_032673
PCGF2	NM_007144
PCGF3	NM_006315
PCGF5	NM_032373
PCGF6	NM_001011663 NM_032154
PJA1	NM_001032396 NM_022368 NM_145119
PJA2	NM_014819
PML	NM_002675 NM_033238 NM_033239 NM_033240 NM_033244 NM_033246 NM_033247 NM_033249 NM_033250
LOC51136	NM_016125
RAD18	NM_020165
ATRX	NM_138271 NM_000489 NM_138270
RBCK1	NM_006462 NM_031229
RAPSN	NM_005055 NM_032645
RAG1	NM_000448
RFPL1	NM_021026
RFPL3	NM_001098535 NM_006604
RFPL4A	XM_292796
RFPL4B	NM_001013734
RBBP6	NM_006910 NM_018703 NM_032626
RC3H1	NM_172071
RC3H2	NM_018835
RCHY1	NM_001008925 NM_001009922 NM_015436
RFFL	NM_001017368 NM_057178
RKHD1	NM_001085363 NM_203304
RKHD2	NM_016626
RKHD3	NM_032246
RKHD4	NM_001093725
RSPRY1	NM_133368
RFWD2	NM_001001740 NM_022457
RFWD3	NM_018124
RNF6	NM_183045 NM_005977 NM_183043 NM_183044
RING1	NM_002931
RNF10	NM_014868
RNF103	NM_005667
RNF11	NM_014372
RNF111	NM_017610
RNF113A	NM_006978
RNF113B	NM_178861
RNF12	NM_016120 NM_183353
RNF121	NM_018320 NM_194452 NM_194453
RNF122	NM_024787
RNF123	NM_022064
RNF125	NM_017831
RNF126	NM_017876 NM_194460
RNF128	NM_024539 NM_194463

RNF13	NM_183382 NM_007282 NM_183381 NM_183383 NM_183384
RNF130	NM_018434
RNF133	NM_139175
RNF135	NM_032322 NM_197939
RNF138	NM_016271 NM_198128
RNF139	NM_007218
RNF14	NM_004290 NM_183398 NM_183399 NM_183400 NM_183401
RNF141	NM_016422
RNF144	NM_014746
RNF145	NM_144726
RNF146	NM_030963
RNF148	NM_198085
RNF149	NM_173647
RNF150	NM_020724
RNF151	NM_174903
RNF152	NM_173557
RNF157	NM_052916
RNF165	NM_152470
RNF166	NM_178841
RNF167	NM_015528
RNF168	NM_152617
RNF169	NM_001098638
RNF17	NM_031277
RNF170	NM_030954
RNF175	NM_173662
RNF180	NM_178532
RNF181	NM_016494
RNF182	NM_152737
RNF183	NM_145051
RNF185	NM_152267
RNF186	NM_019062
RNF187	XM_001129309 XM_928029
RNF19	NM_015435 NM_183419
RNF190	NM_152598
RNF2	NM_007212
RNF20	NM_019592
RNF207	NM_173795 NM_207396
RNF208	NM_031297
RNF212	NM_194439
RNF213	NM_020914
RNF214	NM_001077239 NM_207343
RNF215	NM_001017981
RNF24	NM_007219
RNF25	NM_022453
RNF26	NM_032015
RNF31	NM_017999

RNF32	NM_030936
RNF34	NM_025126 NM_194271
RNF38	NM_022781 NM_194328 NM_194329 NM_194330 NM_194332
RNF39	NM_170770 NM_025236 NM_170769
RNF4	NM_002938
RNF40	NM_014771
RNF41	NM_005785 NM_194358 NM_194359
RNF43	NM_017763
RNF44	NM_014901
RNF5	NM_006913
RNF7	NM_014245 NM_183237
RNF8	NM_003958 NM_183078
RBX1	NM_014248
SIAH1	NM_001006610 NM_003031
SIAH2	NM_005067
SH3RF1	NM_020870
SH3RF2	NM_152550
LOC441061	XM_496738 XM_941081
LOC390231	XM_372423 XM_936301
LOC399937	XM_374917 XM_930197
TRIM49	NM_020358
LOC120824	XM_062300
LOC283116	XM_208043
LOC399939	XM_374919
SHPRH	NM_001042683 NM_173082
SPRYD5	NM_032681
SYVN1	NM_032431 NM_172230
TTC3	NM_001001894 NM_003316
TRAF2	NM_021138
TRAF3	NM_003300 NM_145725 NM_145726
TRAF4	NM_145751 NM_004295
TRAF5	NM_001033910 NM_004619 NM_145759
TRAF6	NM_004620 NM_145803
TRAF7	NM_206835 NM_032271
TOPORS	NM_005802
TRAIP	NM_005879
TRIAD3	NM_207111 NM_207116
TRIML1	NM_178556
TRIM10	NM_006778 NM_052828
TRIM11	NM_145214
TRIM13	NM_001007278 NM_005798 NM_052811 NM_213590
TRIM15	NM_052812 NM_033229
TRIM17	NM_001024940 NM_001024941 NM_016102
TRIM2	NM_015271
TRIM21	NM_003141
TRIM22	NM_006074

TRIM23	NM_001656 NM_033227 NM_033228
TRIM24	NM_003852 NM_015905
TRIM25	NM_005082
TRIM26	NM_003449
TRIM27	NM_030950 NM_006510
TRIM28	NM_005762
TRIM3	NM_006458 NM_033278
TRIM31	NM_052816 NM_007028
TRIM32	NM_012210
TRIM33	NM_015906 NM_033020
TRIM34	NM_001003827 NM_021616 NM_130389 NM_130390
TRIM35	NM_171982
TRIM36	NM_001017397 NM_001017398 NM_018700
TRIM37	NM_001005207 NM_015294
TRIM38	NM_006355
TRIM39	NM_021253 NM_172016
TRIM4	NM_033017 NM_033091
TRIM40	NM_138700
TRIM41	NM_033549 NM_201627
TRIM42	NM_152616
TRIM43	NM_138800
TRIM45	NM_025188
TRIM46	NM_025058
TRIM47	NM_033452
TRIM48	NM_024114
TRIM5	NM_033034 NM_033092 NM_033093
TRIM50	NM_178125
TRIM52	NM_032765
TRIM54	NM_032546 NM_187841
TRIM55	NM_033058 NM_184085 NM_184086 NM_184087
TRIM56	NM_030961
TRIM58	NM_015431
TRIM59	NM_173084
TRIM6	NM_001003818 NM_058166
TRIM60	NM_152620
TRIM61	NM_001012414
TRIM62	NM_018207
TRIM63	NM_032588
TRIM64	XM_061890
TRIM65	NM_173547
TRIM67	NM_001004342
TRIM68	NM_018073
TRIM69	NM_080745 NM_182985
TRIM7	NM_033342 NM_203293 NM_203294 NM_203295 NM_203296 NM_203297
TRIM72	NM_001008274
TRIM73	NM_198924

TRIM74	NM_198853
TRIM75	XM_373039 XM_939332
TRIM8	NM_030912
TRIM9	NM_015163 NM_052978
UBR1	NM_174916
UBR2	NM_015255
UBR4	NM_020765
UHRF1	NM_001048201 NM_013282
UHRF2	NM_152896
UBOX5	NM_014948 NM_199415
UNK	NM_001080419
VPS11	NM_021729
VPS18	NM_020857
VPS41	NM_014396 NM_080631
VPS8	NM_001009921 NM_015303
WHSC1	NM_133336 NM_001042424 NM_007331 NM_133330 NM_133331 NM_133334 NM_133335
ZNRF1	NM_032268
ZNRF2	NM_147128
ZNRF3	NM_032173
ZNRF4	NM_181710
DZIP3	NM_014648
ZNF179	NM_007148
ZNF294	NM_015565
ZNF313	NM_018683
ZNF364	NM_014455
ZNF598	NM_178167
ZNF645	NM_152577
ZNF650	NM_172070
ZFPL1	NM_006782
ZSWIM2	NM_182521
LOC150739	XM_092267
NOSIP	NM_015953
PPIL2	NM_014337 NM_148175 NM_148176
PRPF19	NM_014502
STUB1	NM_005861
UBE4A	NM_004788
UBE4B	NM_006048
UBOX5	NM_014948 NM_199415
WDSUB1	NM_152528
BTRC	NM_003939 NM_033637
CCNF	NM_001761
FBXL10	NM_001005366 NM_032590
FBXL11	NM_012308
FBXL12	NM_017703
FBXL13	NM_145032
FBXL14	NM_152441

FBXL15	NM_024326
FBXL16	NM_153350
FBXL18	NM_024963
FBXL2	NM_012157
FBXL20	NM_032875
FBXL3	NM_012158
FBXL4	NM_012160
FBXL5	NM_012161 NM_033535
FBXL6	NM_012162 NM_024555
FBXL7	NM_012304
FBXL8	NM_018378
FBXW11	NM_012300 NM_033644 NM_033645
FBXW2	NM_012164
FBXW4	NM_022039
FBXW5	NM_018998
FBXW7	NM_001013415 NM_018315 NM_033632
FBXW8	NM_012174 NM_153348
FBXO10	NM_012166
FBXO11	NM_012167 NM_018693 NM_025133
FBXO15	NM_152676
FBXO16	NM_172366
FBXO17	NM_024907 NM_148169
FBXO2	NM_012168
FBXO21	NM_015002 NM_033624
FBXO22	NM_012170 NM_147188
FBXO24	NM_012172 NM_033506
FBXO25	NM_012173 NM_183420 NM_183421
FBXO27	NM_178820
FBXO28	NM_015176
FBXO3	NM_012175 NM_033406
FBXO30	NM_032145
FBXO31	NM_024735
FBXO32	NM_058229 NM_148177
FBXO33	NM_203301
FBXO34	NM_017943
FBXO36	NM_174899
FBXO38	NM_030793 NM_205836
FBXO4	NM_012176 NM_033484
FBXO40	NM_016298
FBXO41	NM_001080410
FBXO42	NM_018994
FBXO44	NM_001014765 NM_033182 NM_183412 NM_183413
FBXO45	XM_931557 XM_946180
FBXO46	NM_001080469
FBXO5	NM_012177
FBXO6	NM_018438

FBXO7	NM_001033024 NM_012179
FBXO8	NM_012180
FBXO9	NM_012347 NM_033480 NM_033481
FBXO18	NM_032807 NM_178150
LRRC29	NM_001004055 NM_012163
RP3-509I19.5	NM_001077706
SKP2	NM_005983 NM_032637
TCEB3	NM_003198
ASB10	NM_080871
ASB11	NM_001012428 NM_080873
ASB13	NM_024701
ASB14	NM_130387
ASB15	NM_080928
ASB16	NM_080863
ASB17	NM_080868
ASB18	NM_212556
ASB2	NM_016150
ASB3	NM_016115 NM_145863
ASB4	NM_016116 NM_145872
ASB5	NM_080874
ASB6	NM_017873 NM_177999
ASB7	NM_024708 NM_198243
ASB8	NM_024095
ASB9	NM_001031739 NM_024087
CISH	NM_145071
NEURL2	NM_080749
RAB40A	NM_080879
RAB40B	NM_006822
RAB40C	NM_021168
LOC286526	NM_001031834
SPSB1	NM_025106
SPSB2	NM_032641
SPSB3	NM_080861
SPSB4	NM_080862
SOCS1	NM_003745
SOCS2	NM_003877
SOCS3	NM_003955
SOCS4	NM_080867 NM_199421
SOCS5	NM_014011 NM_144949
SOCS6	NM_004232
SOCS7	NM_014598
TULP4	NM_001007466 NM_020245
VHL	NM_000551 NM_198156
WSB1	NM_015626 NM_134265
WSB2	NM_018639
ABTB1	NM_032548 NM_172027 NM_172028

ABTB2	NM_145804
ANKFY1	NM_016376 NM_020740
ARMC5	NM_024742
BCL6	NM_001706 NM_138931
BCL6B	NM_181844
BTBD1	NM_001011885 NM_025238
BTBD10	NM_032320
BTBD11	NM_001017523 NM_001018072 NM_152322
BTBD12	NM_032444
BTBD14A	NM_144653
BTBD14B	NM_052876
BTBD2	NM_017797
BTBD3	NM_014962 NM_181443
BTBD6	NM_033271
BTBD7	NM_001002860 NM_018167
BTBD8	NM_183242
BTBD9	NM_152733
BACH1	NM_001011545 NM_001186 NM_206866
BACH2	NM_021813
CCIN	NM_005893
C16orf44	NM_024731
ENC1	NM_003633
LOC388419	NM_001080466
GZF1	NM_022482
GMCL1	NM_178439
GAN	NM_022041
HIC1	NM_001098202 NM_006497
HIC2	NM_015094
LOC339745	NM_001001664
C8ORFK36	NM_001081675
MGC23270	NM_152646
IVNS1ABP	NM_016389 NM_006469
IBTK	NM_015525
IPP	NM_005897
KCNRG	NM_173605 NM_199464
KCTD1	NM_198991
KCTD10	NM_031954
KCTD12	NM_138444
KCTD13	NM_178863
KCTD14	NM_023930
KCTD15	NM_024076
KCTD16	NM_020768
KCTD17	NM_024681
KCTD2	NM_015353
KCTD20	NM_173562
KCTD21	NM_001029859

KCTD3	NM_016121
KCTD4	NM_198404
KCTD5	NM_018992
KCTD6	NM_153331
KCTD7	NM_153033
KCTD8	NM_198353
KCTD9	NM_017634
KCNV1	NM_014379
KCNS1	NM_002251
KCNS2	NM_020697
KCNS3	NM_002252
KCNB1	NM_004975
KCNB2	NM_004770
KCNA1	NM_000217
KCNA10	NM_005549
KCNA2	NM_004974
KCNA3	NM_002232
KCNA4	NM_002233
KCNA5	NM_002234
KCNA6	NM_002235
KCNA7	NM_031886
KCND1	NM_004979
KCND2	NM_012281
KCND3	NM_004980 NM_172198
KCNC1	NM_004976
KCNC2	NM_139136 NM_139137 NM_153748
KCNC3	NM_004977
KCNC4	NM_001039574 NM_004978 NM_153763
KCNG1	NM_002237 NM_172318
KCNG3	NM_133329 NM_172344
KLHDC5	NM_020782
KLHDC6	NM_207335
KBTBD10	NM_006063
KBTBD11	NM_014867
KBTBD2	NM_015483
KBTBD3	NM_152433 NM_198439
KBTBD4	NM_016506 NM_018095
KBTBD5	NM_152393
KBTBD6	NM_152903
KBTBD7	NM_032138
KBTBD8	NM_032505
KLHL1	NM_020866
KLHL10	NM_152467
KLHL11	NM_018143
KLHL12	NM_021633
KLHL13	NM_033495

KLHL14	NM_020805
KLHL15	NM_030624
KLHL17	NM_198317
KLHL18	NM_025010
KLHL20	NM_014458
KLHL21	NM_014851
KLHL22	NM_032775
KLHL23	NM_144711
KLHL24	NM_017644
KLHL25	NM_022480
KLHL26	NM_018316
KLHL28	NM_017658
KLHL29	XM_001134449 XM_940375
KLHL3	NM_017415
KLHL30	NM_198582
KLHL31	NM_001003760
KLHL32	NM_052904
KLHL34	NM_153270
KLHL4	NM_019117 NM_057162
KLHL5	NM_001007075 NM_015990 NM_199039
KLHL6	NM_130446
KLHL7	NM_001031710 NM_018846
KLHL8	NM_020803
KLHL9	NM_018847
KEAP1	NM_012289 NM_203500
LGALS3BP	NM_005567
LZTR1	NM_006767
MYNN	NM_018657
PATZ1	NM_014323 NM_032050 NM_032051 NM_032052
RCBTB1	NM_018191
RCBTB2	NM_001268
RHOBTB1	NM_001032380 NM_014836 NM_198225
RHOBTB2	NM_015178
RHOBTB3	NM_014899
SHKBP1	NM_138392
LOC123103	XM_063481
SPOP	NM_001007226 NM_001007227 NM_001007228 NM_001007229 NM_001007230 NM_00356
TNFAIP1	NM_021137
ZBTB1	NM_014950
ZBTB10	NM_023929
ZBTB11	NM_014415
ZBTB12	NM_181842
ZBTB16	NM_001018011 NM_006006
ZBTB17	NM_003443
ZBTB2	NM_020861
ZBTB20	NM_015642

ZBTB22	NM_005453
ZBTB24	NM_014797
ZBTB25	NM_006977
ZBTB26	NM_020924
ZBTB3	NM_024784
ZBTB32	NM_014383
ZBTB33	NM_006777
ZBTB37	NM_032522
ZBTB38	NM_001080412
ZBTB39	NM_014830
ZBTB4	NM_020899
ZBTB40	NM_001083621 NM_014870
ZBTB41	NM_194314
ZBTB43	NM_014007
ZBTB44	NM_014155
ZBTB45	NM_032792
ZBTB46	NM_025224
ZBTB48	NM_005341
ZBTB5	NM_014872
ZBTB6	NM_006626
ZBTB7A	NM_015898
ZBTB7B	NM_015872
ZBTB7C	NM_001039360
ZBTB8	NM_001040441
ZBTB9	NM_152735
ZNF131	NM_003432
ZFP161	NM_003409
ZNF238	NM_006352 NM_205768
ZNF295	NM_001098402 NM_001098403 NM_020727
ZNF509	NM_145291
FLJ30092	XM_497354 XM_942665
HACE1	NM_020771
HERC1	NM_003922
HERC2	NM_004667
HERC3	NM_014606
HERC4	NM_001017972 NM_015601 NM_022079
HERC5	NM_016323
HERC6	NM_001013000 NM_001013002 NM_001013005 NM_017912
HECTD1	NM_015382
HECTD2	NM_173497 NM_182765
HECTD3	NM_024602
HECW1	NM_015052
HECW2	NM_020760
HUWE1	NM_031407
UBE3A	NM_000462 NM_130838 NM_130839
ITCH	NM_031483

KIAA0317	NM_001039479
KIAA1333	NM_017769
NEDD4	NM_006154 NM_198400
NEDD4L	NM_015277
SMURF1	NM_020429 NM_181349
SMURF2	NM_022739
TRIP12	NM_004238
UBR5	NM_015902
UBE3B	NM_130466 NM_183415
UBE3C	NM_014671
WWP1	NM_007013
WWP2	NM_007014 NM_199423 NM_199424
CPSF1	NM_013291
DDB1	NM_001923
SF3B3	NM_012426
ANUBL1	NM_174890
OTUD7A	NM_130901
OTUD7B	NM_020205
KCTD7	NM_153033
RABGEF1	NM_014504
TNFAIP3	NM_006290
ZFAND3	NM_021943
ZFAND5	NM_006007
ZFAND6	NM_019006

Supplementary Table 2: List of genes for which multiple shRNAs were isolated from the RNAi screen.

Gene Symbol	Gene Name	Protein family
MARCH1	Membrane-associated ring finger (C3HC4) 1	MARCH
MARCH9	Membrane-associated ring finger (C3HC4) 9	MARCH
NHLRC1	NHL repeat containing E3 ubiquitin protein ligase 1	
KCNB1	Potassium voltage-gated channel, Shab-related subfamily, member 1	Potassium voltage-gated channel, Shab-related
RNFT1	Ring finger protein, transmembrane 1	
RNF166	Ring finger protein 166	
DTX3L	Deltex 3-like (Drosophila)	DELTEX

Supplementary Table 3. Primers were used for qRT-PCR analysis, ChIP experiments, and cloning. The antibodies were used as indicated. The source and concentrations of chemical inhibitors and biologics used for drug treatment experiments are summarized.

Application	Gene symbol	Species	Forward Primer (5'-3')	Reverse Primer (5'-3')
**qPCR**	MARCH1	Human	GCCTCACAAACCTCCACATT	TCTGCAGATGTCCTGAGTGG
	MARCH9	Human	TGGAGCTGTGAGCTCTGCTA	CTGAAGGGCTGAGTGAGGAC
	NHLRC1	Human	GTCACCATCACCAACGACTG	TCTCCACACCCCAAGGTAAG
	KCNB1	Human	TGCCAGTGGTGGTAGGTTTG	GAGGAGTGGACTGGGGTCAC
	RNF166	Human	GAAGGTCCAGGAGCAGATGG	TGGCTTTCCACACAGTGCTT
	RNFT1	Human	ATGCCTCAACCCCTCAGTGT	GAGTGACCCCGTAAGCGACT
	DTX3L	Human	TGTCCCACATGCCAGACTTC	TTTCCTGGGTTTGGGTGTTC
	ACTIN	Human	GCATGGAGTCCTGTGGCATC	TTCTGCATCCTGTCGGCAAT
	MARCH1	Mouse	CAGATGACCACGAGCGAAAG	AAGACGAGACCTCCCGTGAA
	MARCH9	Mouse	GTGACGTTCCAGTTCCCACA	TCTGGCATTGGTCTCCCTTT
	NHLRC1	Mouse	GTCACCAACGACTGCCATGT	GCCTCTGCATCAGTCACCAG
	KCNB1	Mouse	GATGGCCAAGACCCAGTCTC	GCTGATGAAGCTGTCGATGC
	RNF166	Mouse	GTGAAGCACTGCGTGGAAAG	CTGGAAGGCAGCTTCTTCGT
	RNFT1	Mouse	CCCACAGTGAAGCAAGACCA	AGCAGCCCAATTCCAAGAGA
	DTX3L	Mouse	GCATCCAGAAAGGGAACCAG	TCGCCGTGTTCCATGATAAG
	ACTIN	Mouse	AGGCTCTTTTCCAGCCTTCC	GTCAGCAATGCCTGGGTACA
	MARCH1	Rat	ACAGGAACCTTGCGCTTTGT	CTTTCGCTCGTGGTCATCTG
	FOXO1	Human	GAGCAGCTGCAATGGCTATG	TGTGTGGGAAGCTTTGGTTG
	CBL	Human	GAGGCCATGGCTCTGAAATC	AGCCGAGCTTTCACTTCGTC
	NEDD4	Human	CCACCAGGTTGGGAAGAAAA	ACCTGCTGGCCTGAATCACT
**ChIP**				

	MARCH1	Human	TTGAGGTCCCCTCGTTGTTT	TGGCTGCTCAGTAGCTCAGG
**Cloning**				
Luciferase reporter	MARCH1 promoter	Human	ATGCGGTACCGCTGAATATTACTCCATTGT	ATGCGTCGACTGACATCTCTCGCCCAGGAG
AAV	MARCH1	Mouse	AGCGGATCCACCATGGATTACAAGGATGACGATGACAAGATGCTGGGCTGGTGTGAAGCG	TCAGACTGATACAACTTCAGG
**Mutagenesis**				
	MARCH1 promoter		CTCAGACTCCCGCCTGTACCAGTACACATCTTTCTCCCT	AGGGAGAAAGATGTGTACTGGGTACAGGCGGGAGTCTGAG
	INSR K1079R		AATGAGGCCTCGGTCATGAGGGGCTTCACCTGCCATCACGTG	CACGTGATGGCAGGTGAAGCCCCTCATGACCGAGGCCTCATT
	MARCH1 Transmembrane I Deletion		ATAATGGAGACCAAGCTCAAATTCATAATGGAGACCAAGCTC	GAGCTTGGTCTCCATTATGAATTTGAGCTTGGTCTCCATTAT
	MARCH1 Transmembrane II Deletion		TTGTATGTATTGATAGACCGGGTGGTAGCCATTGGCTTCACA	TGTGAAGCCAATGGCTACCACCCGGTCTATCAATACATACAA
	MARCH1 shRNA#1 resistant cDNA		AATATTCTGTTCCGTTACCTTTCATGTAATCGCGATCACCT	AGGTGATCGCGATTACATGAAAGGTAACGGAACAGAATATT
	MARCH1 shRNA#2 resistant cDNA		GGAAAAAAAGTAAAATCTCCACTATGTACTACCTTAACCAAGA	TCTTGGTTAAGGTAGTACATAGTGGAGATTTTACTTTTTTTCC
	**Protein symbol**	**Species**	**Antibody, Cat. #**	**Application**
	AKT	Human, Mouse	Cell Signaling #4685	IB
	AKT pS473	Human, Mouse	Cell Signaling #4060	IB
	INSRα	Human, Mouse	Santa Cruz #sc-710	IB
	INSRα	Human, mouse	Pierce #83-14	Flow Cytometry
	INSRβ	Human, Mouse	Cell Signaling #3025	IP, IB

	INSRβ	Human, Mouse	Cell Signaling #3020	IP, IB
	INSRβ	Human, Mouse	Santa Cruz #sc-711	IP, IB
	IRS1	Human, Mouse	Santa Cruz #sc-559	IP, IB
	β-Actin	Human, Mouse	Cell Signaling #3700	IB
	INSR pY1158	Human, Mouse	Cell Signaling #3021	IB
	Na-K ATPase	Human, Mouse	Abcam #ab7671	IB
	pTyr	Human, Mouse	Cell Signaling #8954	IB
	MARCH1	Mouse	Sigma #3D2	IB
	MYC epitope	n/a	Santa Cruz #sc-40	IB
	GFP	n/a	Cell Signaling #2956	IB
	GFP	n/a	Cell Signaling #2955	IB
	GFP	n/a	Santa Cruz #sc-8334	IP, IB
	Flotillin-2	Human	Cell Signaling #3436	IB
	Caveolin-1	Human	Cell Signaling #3267	IB
	Calnexin	Human	Abcam #22595	IB
	FoxO1	Human	Cell Signaling #2880	ChIP
	Ubiquitin	Multiple species	Biomol #PW8810	IP, IB
	HA-Tag	n/a	Cell signaling #3724	IP, IB
	**Inhibitor**		**Source**	
	Cycloheximide	100µg/ml	Sigma #C1988	
	Cytochalasin B	5 µM	Sigma #C6762	
	PTP1B inhibitor	10µM	Calbiochem #539749	
	Dynasore	20µM	Calbiochem # 324410	
	**Chemicals /Biologics**			
	Apo Transferrin	5 µg/ml	Sigma #T1147	
	Dexamethasone	0.25 µM	Sigma #D4902	
	EGF	50 ng/ml	Sigma #E9644	
	FGF	10 ng/ml	Sigma #SRP3043	
	Fibronectin	0.5µg/ml	Sigma, #F1141	
	Hydrocortisone	100nM	Sigma #H0888	
	Insulin	0.05µg/ml	Sigma #D4902	

	Isobutylmethylxanthine	0.5 mM	Sigma #I7018	
	N-ethyl maleimide	2mM	Sigma #3876	
	Selenium	5ng/ml	Sigma #S5261,	
	USP2	1μM	Boston Biochem #E506	

Supplementary Table 4. shRNA IDs for the shRNAs used in this study

Gene Name	Species	shRNA IDs	
		**shRNA#1**	**shRNA#2**
MARCH1	Human	TRCN0000037019	TRCN0000037022
MARCH9	Human	TRCN0000073170	TRCN0000073171
NHLRC1	Human	TRCN0000034184	TRCN0000034186
KCNB1	Human	TRCN0000044783	TRCN0000044785
RNF166	Human	TRCN0000007801	TRCN0000007803
RNFT1	Human	TRCN0000034332	TRCN0000034333
DTX3L	Human	TRCN0000073208	TRCN0000073210
FOXO1	Human	TRCN0000039578	TRCN0000010333
CBL	Human	TRCN0000039723	TRCN0000039724
NEDD4	Human	TRCN0000007553	TRCN0000007554
March1	Mouse	V3LMM_461750	TRCN0000176313
